# Effect of rainfall on metagenomics in a sewage environment in Hongta District, Yuxi city, Yunnan Province

**DOI:** 10.7717/peerj.20199

**Published:** 2025-11-19

**Authors:** Senquan Jia, Wenpeng Gu, Lili Jiang, Yong Zhang, Xiaoqing Fu, Jianwen Yin, Yongming Zhou

**Affiliations:** 1 Institute of Acute Infectious Disease Control and Prevention, Yunnan Provincial Centers for Disease Control and Prevention, Kunming, China; 2 Yunnan International Joint Laboratory for Public Health and Disease Control, Kunming, China; 3 Yunnan Key Laboratory of Cross-Border Infectious Disease Control and Prevention and Novel Drug Development, Kunming, China

**Keywords:** Hongta District, Yuxi city, Sewage, Metagenomics, ARGs

## Abstract

**Background:**

Hongta District of Yuxi city is located in the central region of Yunnan Province, Southwest China. Previous studies have shown a high prevalence of enteric infectious diseases in the area, which may be related to sewage discharge. However, there has been no systematic analysis of the microbiome in sewage in this area. In this study, we investigated environmental sewage in Hongta District, Yuxi city, Yunnan Province.

**Methods:**

Surveillance was conducted in Hongta District, Yuxi city, for a period of one year. At both its urban and rural sites, sewage samples were collected for metagenomic sequencing.

**Results:**

The results revealed that in the sewage samples, bacteria accounted for 98.31% of the total microbiome, followed by Archaea (1.05%), Viruses (0.30%) and Eukaryota (0.34%). At the phylum level, Proteobacteria was the taxon with the highest relative abundance, accounting for 57.57% of all samples, followed by Firmicutes (17.17%), Bacteroidetes (12.23%), Actinobacteria (7.10%), and Synergistetes (1.45%). At the genus level, the taxa with the highest relative abundances of all the microbiomes were *Acidovorax* (6.63%), *Pseudomonas* (4.98%), *Acinetobacter* (4.23%), *Comamonas* (3.85%), and *Aliarcobacter* (2.78%). The diversity of the samples grouped by site and rainfall formed their own clusters, but only the compositions of different taxa grouped by rainfall significantly differed (*P* = 0.038 at the family, *P* = 0.019 at the genus and *P* = 0.005 at the species level). In general, the abundance of several taxa at the family, genus and species levels in the dry season group was higher (*P* < 0.05) than that in the rainy season group according to the Kruskal–Wallis test. The relative abundance s of most virulence genes were higher at urban sites than at rural sites, while those in the rainy season was higher than those in the dry season. The distribution of antibiotic resistance genes (ARGs) in urban and rural sewage was significantly different (*P* = 0.018). The relative abundance of multidrug resistance genes in urban sewage was higher than that in rural sewage, and the relative abundance of most resistance genes in the dry season group was higher than that in the rainy season group.

**Conclusions:**

In general, the abundance and distribution features of the sewage microbial communities in the Hongta District of Yuxi city were affected by site and rainfall factors, with significant regional and temporal specificity. Strengthening the surveillance of environmental sewage and improving discharge methods are highly important for ensuring public health security.

## Introduction

The spread and prevalence of infectious diseases are closely related to the discharge of pollutants, among which the discharge and circulation of sewage are important factors (Schneider et al., 2021). The biological treatment of wastewater is driven by mixed microbial communities, and the transformation and elimination of pollutants depend on the physiological processes of microorganisms (Steen et al., 2019). Sewage mainly contains two types of pollutants: chemical pollutants and biological pollutants. Among them, biological pollutants include pathogenic bacteria, viruses, toxic algae and antibiotic resistance genes (ARGs) (Apreja et al., 2022; Larsson & Flach, 2022). In recent years, the types and emissions of biological pollutants have been increasing, as have the requirements and difficulty of their detection. Metagenomic sequencing technology can detect changes in the abundance of all organisms in a specific environment and is an ideal solution for sewage surveillance (Blow, 2008; Hugenholtz & Tyson, 2008).

With the continuous development of urbanization and industrialization in China, the volume of sewage discharge has increased, and the contamination and potential risks of pathogenic microorganisms in sewage treatment have also increased (Chen et al., 2023; Zhao et al., 2024). With the continuous improvement in rural living standards, domestic sewage has doubled and become an important source of surface water and soil (Zhang et al., 2022). Hongta District of Yuxi city, is located in the central part of Yunnan Province (Liu et al., 2024). The socioeconomic development model involves mixed urban and rural development, with both urban areas having relatively concentrated populations and scattered rural areas. Yuxi city is an area with a high incidence of infectious diseases in Yunnan Province, especially the occurrence and prevalence of intestinal infectious diseases (Gu et al., 2015). Systematic research and analysis on environmental sewage in this area have not been previously conducted. Therefore, the Hongta District of Yuxi city was selected for metagenomic sequencing and analysis of sewage samples to determine the characteristics and influencing factors of the sewage microbial communities in this area.

## Materials and Methods

### Sampling sites and sample collection

We carried out one year of environmental sewage surveillance in Hongta District of Yuxi city, central Yunnan Province. Fifteen environmental sewage sampling sites were selected for the study, covering the entire Hongta District of Yuxi city. The sample collection locations included rural environmental sewage and urban environmental sewage, ditch water and river drainage. The data were collected once a month from March 2023 to March 2024. A 500 mL sample was collected at each sampling point. The collected samples were tested for microbial indicators such as fecal contamination indicators of bacteria.

From the 15 sampling sites, two sewage sample collection sites were selected as the research objects (the urban sewage point, and the rural sewage point). The samples were enriched *via* centrifugation (4,000 ×g, 4 °C, centrifuged for 10 min), and the centrifuged sediment was used for metagenomic sequencing. A total of 24 samples were subjected to DNA extraction and metagenomic sequencing.

Statistics were performed by two grouping factors. One was the sampling sites, namely the urban and the rural areas. Another grouping factor was the rainfall in Hongta District of Yuxi city. We used the rainy seasons of June, July, August and September in the Hongta District of Yuxi city as the rainy season group. During these four months, the average rainfall for each month was higher than 100 mm. The remaining months, were included in the dry season group. During these months, the average rainfall in China is less than 100 mm each month (Xia et al., 2020). The details of the samples are shown in Table S1.

### Metagenomics

Genomic DNA was extracted *via* a soil sample DNA extraction kit (Tiangen, Beijing, China) according to the manufacturer’s instructions. The DNA quality was detected *via* the use of a Qubit 2.0 instrument (Thermo Fisher Scientific) and a Nanodrop. Qualified genomes were fragmented to five bp and then end-repaired, A-tailed, and adapter ligated *via* the NEB Next DNA Library Prep Kit for Illumina (NEB, Ipswich, MA, USA) according to the instructions. DNA fragments with lengths of 300–400 bp were enriched *via* PCR and purified *via* the AMPure XP system (Beckman Coulter, Brea, CA, USA). The libraries were analyzed for size distribution *via* a 2100 Bioanalyzer (Agilent, Santa Clara, CA, USA) and quantified *via* real-time PCR. Finally, genome sequencing was performed on the Illumina NovaSeq 6000 platform with a paired-end 150 (PE150) reagent kit (Liao et al., 2023).

Raw data were filtered *via* FASTP (version 0.18.0). Clean reads of each sample were assembled individually *via* MEGAHIT (version 1.1.2) with steps over a k-mer range of 21-141 to generate sample (or group)-derived assemblies (Li et al., 2015). Genes were predicted on the basis of the final assembly contigs (>500 bp) *via* MetaGeneMark (version 3.38). The reads were aligned to predict genes *via* Bowtie (version 2.2.5) to count the number of reads (Langmead & Salzberg, 2012). The final gene catalog was obtained from nonredundant genes with gene read counts >2. The plots were graphed *via* the R gplot2 package. The unigenes were annotated *via* DIAMOND (version 0.9.24) by alignment with the deposited unigenes in different databases, including Nr, KEGG, eggNOG, CAZy, PHI, VFDB, and CARD (Buchfink, Xie & Huson, 2015).

The Chao1, ACE, Shannon, and Simpson indices were calculated *via* the Python package (version 0.5.6). Alpha indices were compared between groups *via* the Wilcoxon rank test. A Bray-Curtis distance matrix based on gene/taxon/function abundance was generated *via* the R Vegan package. Multivariate statistical techniques, including principal component analysis (PCA), principal coordinates analysis (PCoA) and non-metric multidimensional scaling (NMDS), were calculated and plotted *via* the R ggplot2 package (Chen & Boutros, 2011). Analysis of similarity (ANOSIM) tests were performed *via* the R project Vegan package. To screen for species biomarkers with significant differences between groups, the rank sum test was used to detect the differential species between different groups, dimensionality reduction was achieved, and the influence of the differential species was evaluated *via* linear discriminant analysis (LDA) to obtain the LDA score. On the basis of KEGG and published literature information, marker gene sets for carbon fixation (137 KOs), the nitrogen cycle (32 KOs), the phosphorus cycle (53 KOs), and the sulfur cycle (88 KOs) were constructed. By extracting the above information from the functional annotation results of the database, and on the basis of the transcripts per million (TPM) abundance calculation method, we performed functional composition analysis, difference analysis and correlation analysis. The corrections for multiple comparisons were performed in the R package using FDR adjustment (false discovery rate) for the number of comparisons. All metagenomic analysis codes are deposited in Data S1.

### Data availability

All data generated or analyzed during this study were included in this article. The sequence data have been deposited into the National Center for Biotechnology Information (NCBI) with BioProject accession number: PRJNA1138443.

## Results

### Quality control and assembly statistics

The quality control results of the raw data revealed that the average number of sequencing reads for all 24 samples was 82,234,844 ± 1,380,6826.6, the percentage of clean reads was 98.99% ± 0.26%, the average GC content was 48.42% ± 4.54%, and the average value of the Q30 was 94.42% ± 0.53%, as shown in Table S2.

The data assembly results revealed that the average ORF value was 530,385 ± 124,805.65, the assembly contig values were 339,444 ± 47,295.67, and the average contig length was 1,031.80 ± 86.23, as shown in Table S3.

### Microbiome composition and abundance analysis

The number of unique genes obtained *via* gene prediction was 3,522,427. The clustering results of gene abundance correlations between samples are shown in Fig. S1A. The shared and unique gene information among the different samples is shown in Fig. S1B. The number of core genes in all the samples was 50,262, the minimum number of genes was 943,752 (HT0808), and the maximum number of annotated genes was 1,471,387 (HT1108).

**Figure 1:**
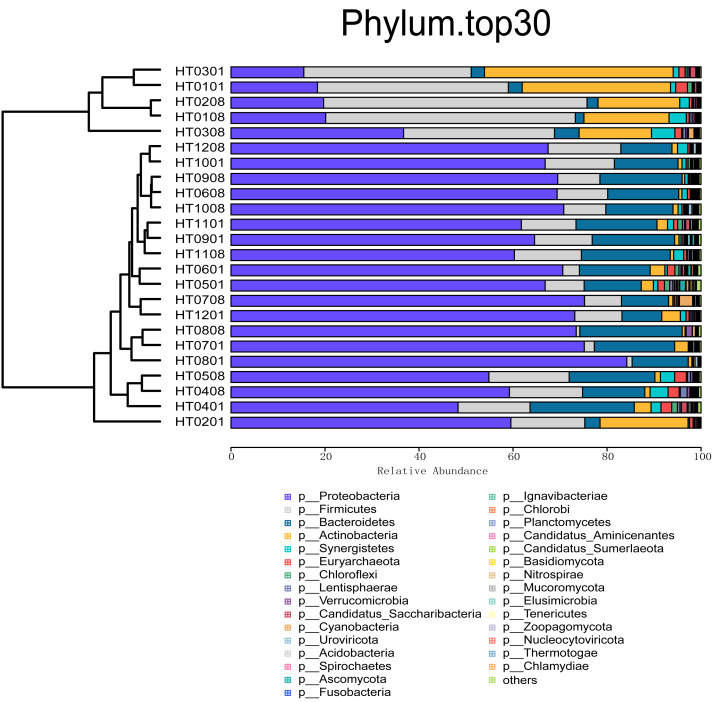
Cluster analysis of abundance at the phylum level and stacking plot of composition.

**Figure 2:**
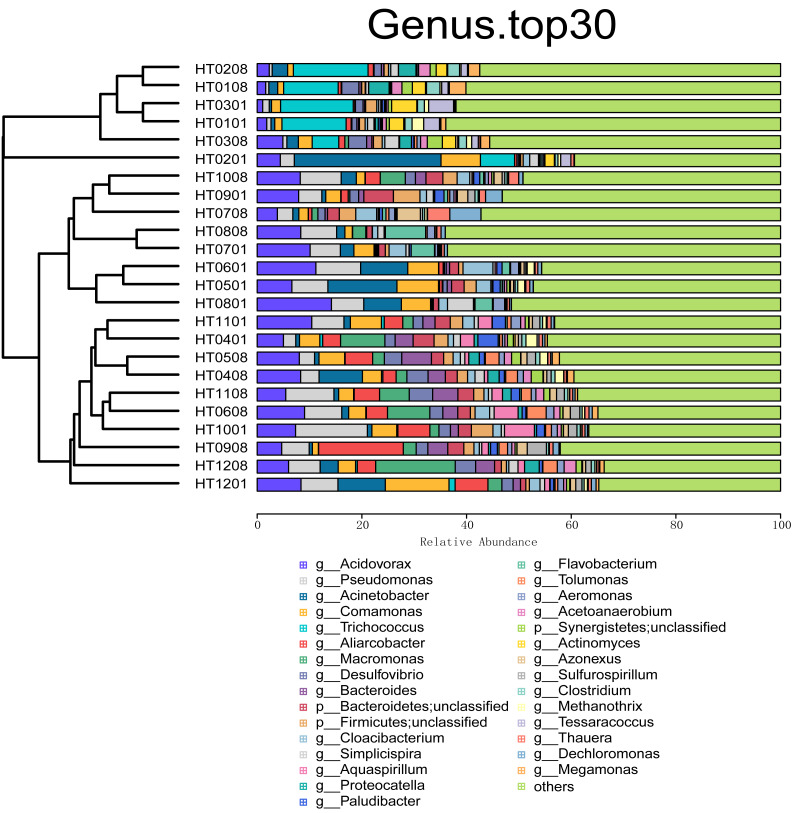
Cluster analysis and stacking diagram at the genus level.

On the basis of the relative abundance information of all samples at different taxon levels (the top 30 in terms of overall abundance were selected), the Bray distance between the samples was calculated, and hierarchical clustering was subsequently performed on the basis of the Bray distance. At the kingdom taxonomic level, bacteria accounted for 98.31% of all microbiome annotations. Archaea accounted for 1.05%, and viruses and Eukaryota accounted for 0.30% and 0.34%, respectively. At the phylum level, Proteobacteria was the taxon with the highest relative abundance, accounting for 57.57% of all samples, followed by Firmicutes (17.17%), Bacteroidetes (12.23%), Actinobacteria (7.10%), and Synergistetes (1.45%), as shown in Fig. 1. At the order level, the taxa with the highest relative abundances were Burkholderiales (22.83%), Eubacteriales (8.49%), Bacteroidales (5.77%), Pseudomonadales (4.59%), and Rhodocyclales (4.25%). At the class level, the taxa with the highest relative abundances were Betaproteobacteria (30.38%), Gammaproteobacteria (16.02%), Clostridia (8.39%), Actinomycetia (6.71%), and Bacteroidia (6.14%). At the family level, the taxa with the highest relative abundances were *Comamonadaceae* (17.71%), *Pseudomonadaceae* (4.76%), *Moraxellaceae* (4.41%), *Arcobacteriaceae* (3.50%), and *Carnobacteriaceae* (2.60%). At the genus level, the taxa with the highest relative abundances were *Acidovorax* (6.63%), *Pseudomonas* (4.98%), *Acinetobacter* (4.23%), *Comamonas* (3.85%), and *Aliarcobacter* (2.78%) (Fig. 2). At the species level, the taxa with the highest relative abundances were *Macromonas bipunctata* (2.99%), *Acidovorax temperans* (2.67%), *Cloacibacterium normanense* (1.89%), *Firmicutes bacterium* (1.63%), and *Acidovorax caeni* (1.49%).

### Diversity analysis

**Figure 3:**
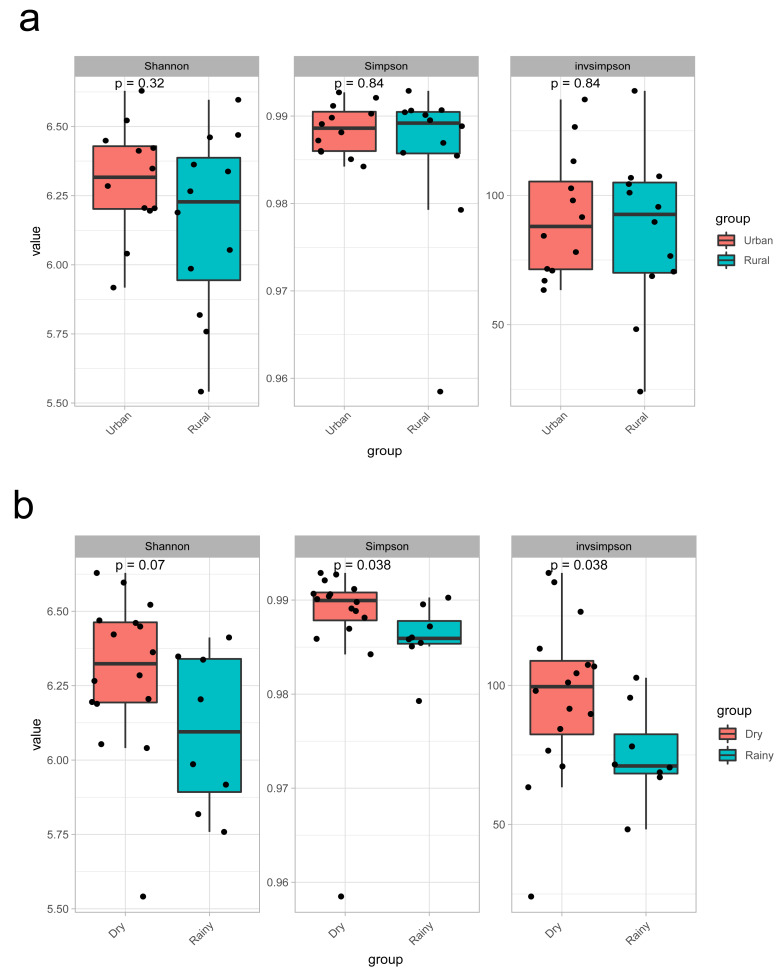
(A–B) Diversity results in this study.

The results of α diversity grouped by sampling site revealed that the Shannon index, Simpson index, and invsimpson index were not significantly different (*P* > 0.05) (Fig. 3A). However, the α diversity results grouped according to the rainfall indicator revealed no significant difference in the Shannon index (*P* = 0.07), whereas the Simpson index and invsimpson index were significantly different (*P* = 0.038) (Fig. 3B).

**Figure 4:**
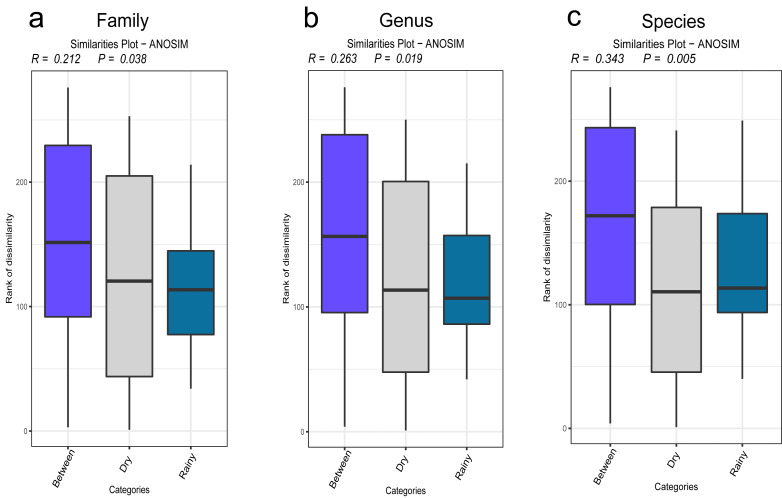
(A–C) Statistics of the diversity results.

The results of the β diversity analysis revealed that, on the basis of the PCA of the sampling sites, the urban and the rural areas formed two clusters at the family, genus and species levels. However, ANOSIM revealed that at any taxonomic level, the compositions of the urban and the rural areas were not significantly different (*P* > 0.05). The results according to the rainfall revealed that at the family, genus, and species levels, the PCA results also formed two clusters (rainy group and dry group). The ANOSIM analysis revealed that the results by the rainfall were significantly different at the family, genus, and species levels (Figs. 4A–4C). The corresponding *P* values were 0.038, 0.019 and 0.005, respectively.

### Differential taxa features and functional annotations

The difference in the sewage microbiome grouped by the rainfall was statistically significant. We conducted a Kruskal–Wallis (KW) rank test on the differential microbial communities at different taxonomic levels, such as family, genus, and species. The results revealed that at the family level, the relative abundances of *Carnobacteriaceae*, *Actinomycetaceae*, *Lachnospiraceae*, *Clostridiaceae*, and *Bifidobacteriaceae* in the dry group were significantly higher than those in the rainy group (Fig. S2). At the genus level, the relative abundances of *Actinomyces*, *Eubacterium*, *Romboutsia*, and *Corynebacterium* in the dry group were higher than those in the rainy group (Fig. S3). At the species level, the relative abundances of *Romboutsia timonensis*, *Eubacterium aggregans*, *Dialister invisus*, and *Eubacterium barkeri* were higher in the dry group than in the rainy group (Fig. S4). Overall, the microbiome abundances of the dry group at different taxonomic levels were almost all higher than that of the rainy group, indicating that the microbial communities in the sewage during the dry season were generally higher than those in the sewage samples during the rainy season.

To screen for biomarkers with significant differences between groups, we performed linear discriminant analysis (LDA) and obtained the LDA score. The biomarkers at different taxon levels according to an LDA score greater than 3 are displayed. The results revealed that at the family level, the dry group generated 27 markers, whereas the biomarker of the rainy group was 17 (Fig. S5). At the genus level, there were 30 biomarkers in the dry and 31 biomarkers in the rainy group (Fig. S6). At the species level, there were 25 biomarkers in the dry group and 36 biomarkers in the rainy group (Fig. S7).

The results of the VFDB virulence factor annotation of the sewage sample revealed that the relative abundance of several virulence genes differed across the sampling sites. Most of the virulence genes presented higher relative abundances at urban sites than at rural sites. Genes, such as *flhA*, *crc*, *fliA*, *waaF*, *fleQ* and *algU* presented relatively high relative abundances at the Urban site. Only a few virulence genes, such as *katB* and *flmH*, had relatively high relative abundances in rural areas. However, after adjusting the *P*-values, no statistically significant differences were found. Analysis of virulence genes based on rainfall showed that the relative abundance of most of the virulence genes was higher in the rainy season than in the dry season, especially *tufA*, *fliI*, *fliM*, *fliN*, *flgI* and *carB*. The relative abundance of few virulence genes was higher in the dry season than in the rainy season, such as *clpP*, *cps4L* and *groEL*. However, similar to the aforementioned statistical results, all *P*-values showed no statistically significant difference after adjustment.

### Annotation analysis of biogeochemical cycles

The Kyoto Encyclopedia of Genes and Genomes (KEGG) functional enrichment of the biogeochemical cycles revealed that the carbon, nitrogen and phosphorus cycles were significantly different among the rainfall groups, with *P* values of 0.012, 0.031 and 0.019, respectively, as shown in Figs. S8A–S8C. On the basis of the gene set screened from the metagenomic data and the information corresponding to the gene set, we analyzed the contribution of taxa to function-specific genes and visually displayed the results in a stacked column graph. Among the genera, different taxa in the dry group and the rainy group contributed differently to the different stages of the carbon cycle. Taxa such as *Trichococcus*, *Eubacterium*, *Methanobacterium* and *Methylocystis* contributed more to the dry group, whereas *Methylomagnum*, *Methanothrix* and *Pseudomonas* contributed more to the rainy group. In terms of genera and taxa, the contributions of different taxa in the dry group and the rainy group to the different stages of the nitrogen cycle differed. Taxa such as *Desulfovibrio*, *Dechloromonas*, *Aliarcobacter* and *Trichococcus* contributed more strongly to the dry group, whereas *Methylomagnum*, *Geobacter*, and *Pseudomonas* contributed more strongly to the rainy group. Among the genera, different species in the dry group and the rainy group contributed differently to the different stages of the phosphorus cycle. *Desulfovibrio*, *Acinetobacter*, and *Aliartobacter* contributed more strongly to the dry group, whereas *Acidovorax*, *Azospira*, and *Pseudomonas* contributed more strongly to the rainy group.

### ARG analysis

**Figure 5:**
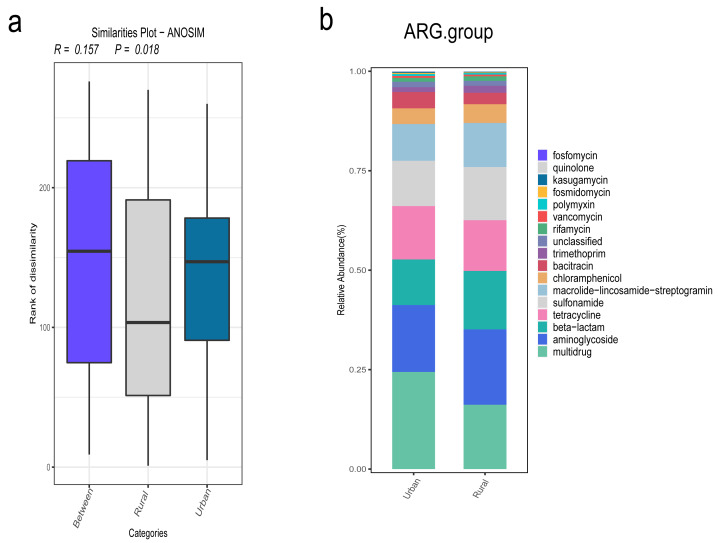
(A–B) Comparison of antibiotic resistance gene annotations and relative abundances.

**Figure 6:**
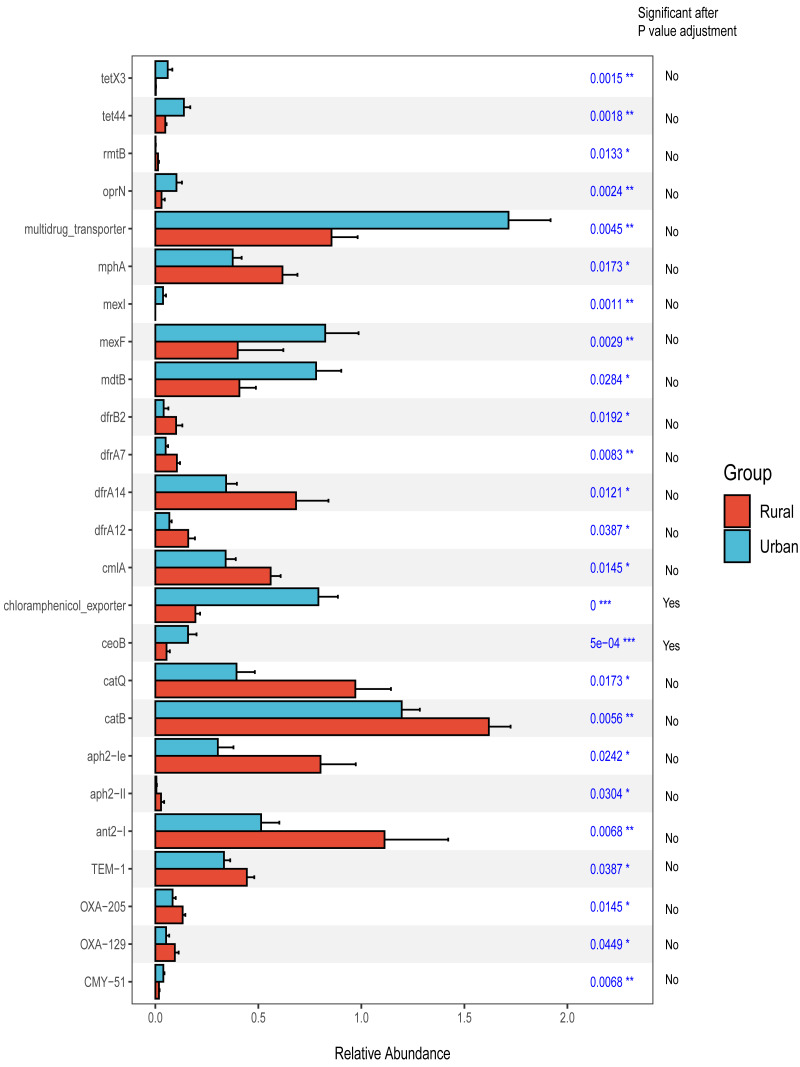
Wilcoxon test of differential antibiotic resistance genes grouped by sampling site.

**Figure 7:**
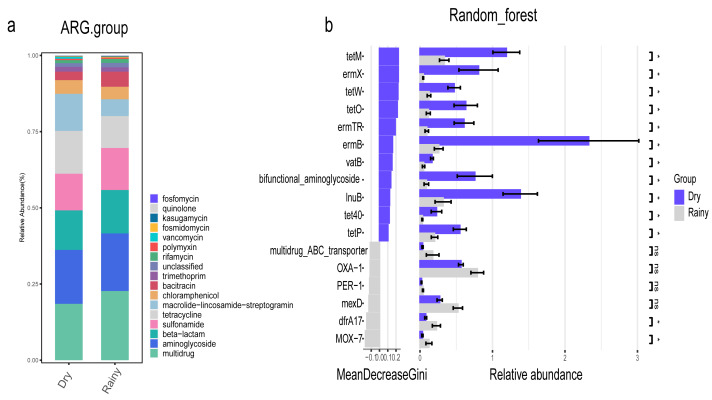
Relative abundance and statistics of ARGs.

In addition, we focused on the distribution characteristics of antibiotic resistance genes in environmental sewage from the Hongta District of Yuxi city. The distributions of the ARG genes in urban and rural areas significantly differed by region (*P* = 0.018), as shown in Fig. 5A. In other words, the distributions of antibiotic resistance genes in rural wastewater and urban wastewater differ. The relative abundance of multidrug resistance genes in the urban group was higher than that in the rural group, whereas the relative abundance of resistance genes such as beta-lactam, aminoglycoside, macrolide-lincosamide-streptogramin, and sulfonamide genes was higher in the rural group (Fig. 5B). The results of the Wilcoxon test revealed that the abundance of two drug resistance genes, chloramphenicol_exporter and *ceoB*, in the urban group was higher than that in the rural group after *P*-values adjustment (Fig. 6). The distributions of the contribution of *Escherichia* to aminoglycoside resistance, the contribution of *Aeromonas* to beta-lactams, and the contribution of *Pseudomonas* to tetracycline resistance differed between the urban and rural groups. Similarly, we analyzed the effects of rainfall on the generation of ARGs, and the results were shown in Fig. 7A. The relative abundances of macrolide-lincosamide-streptogramin and tetracycline in the dry group were higher than those in the rainy group, whereas the relative abundances of multidrug and bacitracin were higher in the rainy group. The results of random forest analysis revealed that the relative abundance of most drug resistance genes in the dry group was higher than that in the rainy group (Fig. 7B), such as tet, erm, vat, bifunctional aminoglycoside, *etc.* In the dry group and the rainy group, the contributions to resistance to different antibiotics also included their own characteristic flora.

## Discussion

The sediment after sewage enrichment is mostly formed by various types of microorganisms, such as prokaryotes, fungi, micro-animals (protozoa and metazoan), and viruses, mixed with extracellular polymers, suspended particulate matter and dissolved organic matter in wastewater (Flach et al., 2021; Imchen et al., 2022). The sewage prokaryotic community is composed of two groups, bacteria and archaea (Ettwig et al., 2010). The results based on the 16S rRNA amplicon revealed that the bacteria belonged to at least 30-40 phyla and more than 700 genera (Ju & Zhang, 2015). Key pollution and purification functions, such as organic matter degradation, nitrification, and biological phosphorus removal, are determined by bacterial communities, whereas community diversity and composition are driven by both environmental and biotic factors (Miao & Liu, 2018). Recently, a study on the global diversity and biogeography of bacterial communities in activated sludge wastewater treatment plants revealed that the spatial turnover rate of microbial communities was related to the scale of the study; however, microbial community construction seemed to be largely driven by stochastic processes (such as dispersal and drift) (Shi et al., 2023).

Our results revealed that bacteria were the absolute number of taxa present in the sewage samples. At the genus level, *Acidovorax*, *Pseudomonas*, *Acinetobacter*, *Comamonas*, and *Aliarrobacter* were the taxa with the highest relative abundances in the sewage samples. *Acidovorax* and *Acinetobacter* are both gram-negative bacteria. The first of these two functions is organic matter degradation: some *Acidovorax/Acinetobacter* bacteria have the ability to decompose organic matter, including polycyclic aromatic hydrocarbons, organic waste and other refractory organic matter. The second is the nitrogen cycle: *Acidovorax/Acinetobacter* can participate in the nitrification and denitrification processes of nitrogen in the soil, thus helping to provide the nitrogen source required by plants. The third is beneficial reciprocity: certain *Acidovorax/Acinetobacter* species establish a symbiotic relationship with plant roots to help plants absorb insoluble mineral nutrients, such as iron and phosphorus, by secreting dissolved organic matter (such as extracellular polymers and enzymes) (Cao et al., 2023; Heylen, Lebbe & De Vos, 2008). Therefore, the characteristics of the microbial communities in environmental wastewater are closely related to their metabolism and function.

On the other hand, the collection of rainwater and sewage in natural ditches or open channels is affected by rainfall, which in turn has a significant effect on changes in microbial communities. Allsing et al. (2022) analyzed the microbial contamination in the US portion of the Tijuana River. They found a pathogen profile of the most abundant disease-causing microbes and viruses present in each of the samples. The specific markers of fecal contamination were identified and linked to each site. The diversity analysis between the sites showed clear distinction as well as similarities between sites and dates, and antibiotic-resistant genes were found at each site (Allsing et al., 2022). In this study, the abundance of the dry group at different taxonomic levels was higher than that of the rainy group, indicating that the abundance of taxa at different levels in the wastewater during the dry season was generally higher than that in the wastewater samples during the rainy season. During the rainy season, rainwater washes away a large number of pollutants from the surface, including various pathogenic microorganisms, which are collected in the wastewater drainage system. Therefore, the abundance of pathogenic microorganisms in microbial communities during the rainy season increased, and the corresponding probability of disease occurrence also increased, especially the occurrence and prevalence of intestinal infectious diseases. Part of the farmland irrigation water in this area comes from the sewage drainage system, which greatly increases the chance of the incidence of infectious diseases, especially those caused by crops such as raw vegetables and fruits (Gu et al., 2015). Therefore, the threat of wastewater discharge during the rainy season to public health has increased significantly. In contrast, in the dry season, because there was less water in the sewage drainage system, the pollutants in the wastewater were concentrated, and the abundance of the microbial communities was higher than that in the rainy season. The drainage methods of rainwater and sewage are significantly influenced by natural factors, which is a link that urgently needs to be improved in the process of urbanization.

A critical step against global antimicrobial resistance (AMR) is effective and accurate surveillance (Apreja et al., 2022; Aslam et al., 2018; Larsson & Flach, 2022). However, at present, antibiotic resistance surveillance relies mainly on the voluntary and passive reporting of a relatively small number of clinical isolates, and reporting often lags behind. Hendriksen et al. (2019) used metagenomic technology to perform resistance analysis on untreated sewage from 79 locations in 74 cities in 60 countries from seven regions, demonstrating the potential application value of this supplementary monitoring method. Analysis of gene sequences revealed that the average level of AMR genes was 0.03%. The researchers obtained samples on different dates at each of the eight locations, which demonstrated a high degree of within-site repeatability. A comparison of samples from different countries showed that “a single sample collected in a large city can represent the overall AMR level in the country.” Among the 1,546 samples of bacterial genera, many bacteria came from feces, but the presence of other genera suggested that the sources were from the environment. Furthermore, these municipal wastewater samples were more similar to the human fecal microbiome than to the microbiomes in the feces of chickens, pigs, or mice. The abundance of AMR genes varied across locations and continents, with the highest level occurring in Africa. Brazil ranked the highest among all countries. Oceania had the lowest abundance of AMR genes. The researchers detected a total of 1,625 AMR genes in 408 genomes, including newly discovered genes such as *CTX-M*, *NDM*, *mcr*, and *optrA*. The most abundant genes were genes encoding resistance genes to macrolides, tetracyclines, aminoglycosides, β-lactams and sulfa drugs. Most European and North American samples contained abundant macrolide resistance genes, and African and Asian samples presented relatively abundant sulfa and phenolic resistance genes. A total of 15 AMR genes accounted for more than 50% of the total abundance of AMRs.

The distributions of antibiotic resistance genes in rural wastewater and urban wastewater were different. The abundance of multidrug resistance genes in urban wastewater was significantly higher than that in rural areas, which was closely related to human settlement density and activities. The environment and distribution of ARG resistance in animals were closely related to human activities, as confirmed by our previous studies. In addition, ARGs were also correlated with rainfall. The relative abundance of most antibiotic resistance genes in the dry season group was higher than that in the rainy season group. Fierer et al. (2022) analyzed the spatial and temporal changes in sewage microbiomes across a university campus. They concluded that sewage systems harbor extensive microbial diversity, including microbes derived from both human and environmental sources. In conclusion, strengthening the monitoring and analysis of wastewater was of great public health importance in terms of environmental pollution and the incidence of disease in the region.

## Conclusions

In this study, we performed metagenomic sequencing for sewage monitoring in Hongta District, Yuxi city, Yunnan Province. The abundance and distribution features of the sewage microbial communities in the Hongta District of Yuxi city were affected by site and rainfall factors, with significant regional and temporal specificity. Therefore, strengthening the monitoring of environmental sewage and improving discharge methods are highly important for ensuring public health security.

##  Supplemental Information

10.7717/peerj.20199/supp-1 Supplemental Information 1 Sample correlation and gene numbers of samples

10.7717/peerj.20199/supp-2 Supplemental Information 2 Statistical analysis of taxa differences at the family level according to the rainfall

10.7717/peerj.20199/supp-3 Supplemental Information 3 Statistical analysis of taxa differences at the genus level according to the rainfall

10.7717/peerj.20199/supp-4 Supplemental Information 4 Statistical analysis of taxa differences at the species level according to the rainfall

10.7717/peerj.20199/supp-5 Supplemental Information 5 LDA analysis at the family level

10.7717/peerj.20199/supp-6 Supplemental Information 6 LDA analysis at the genus level

10.7717/peerj.20199/supp-7 Supplemental Information 7 LDA analysis at the species level

10.7717/peerj.20199/supp-8 Supplemental Information 8 KEGG functional enrichment results of biogeochemical cycles

10.7717/peerj.20199/supp-9 Supplemental Information 9 All metagenomic analysis codes

10.7717/peerj.20199/supp-10 Supplemental Information 10 The sample details in this study

10.7717/peerj.20199/supp-11 Supplemental Information 11 Data quality control results for all sequencing samples

10.7717/peerj.20199/supp-12 Supplemental Information 12 Assembly statistics for the sequencing data

10.7717/peerj.20199/supp-13 Supplemental Information 13 Codebook

10.7717/peerj.20199/supp-14 Supplemental Information 14 Application of FDR Correction in Metagenomic Analysis

10.7717/peerj.20199/supp-15 Supplemental Information 15 R Package Data Visualization and Statistical Analysis Code

10.7717/peerj.20199/supp-16 Supplemental Information 16 Metagenomic Analysis Workflow

## Additional Information and Declarations

### Data Availability

The following information was supplied regarding data availability: The data is available at NCBI BioProject: PRJNA1138443. The code is available in the Supplemental File.
