# Supplementary figures and images for: Effect of rainfall on metagenomics in a sewage environment in Hongta District, Yuxi city, Yunnan Province
Source: PeerJ. 2025 Nov 19;13:e20199. doi: 10.7717/peerj.20199 (PMC12640135; doi:10.7717/peerj.20199)

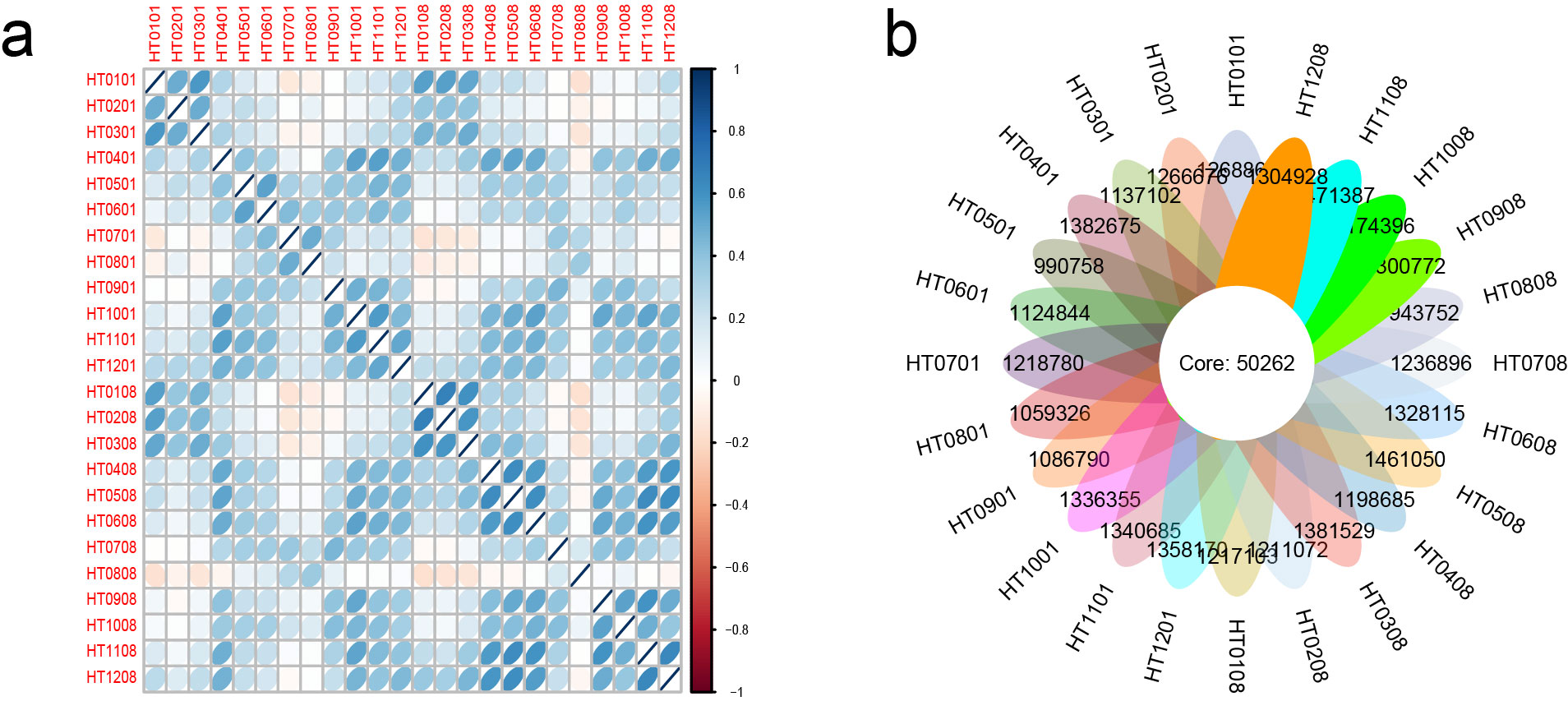

Supplement: Supplemental Information 1 [file peerj-13-20199-s001.jpg]

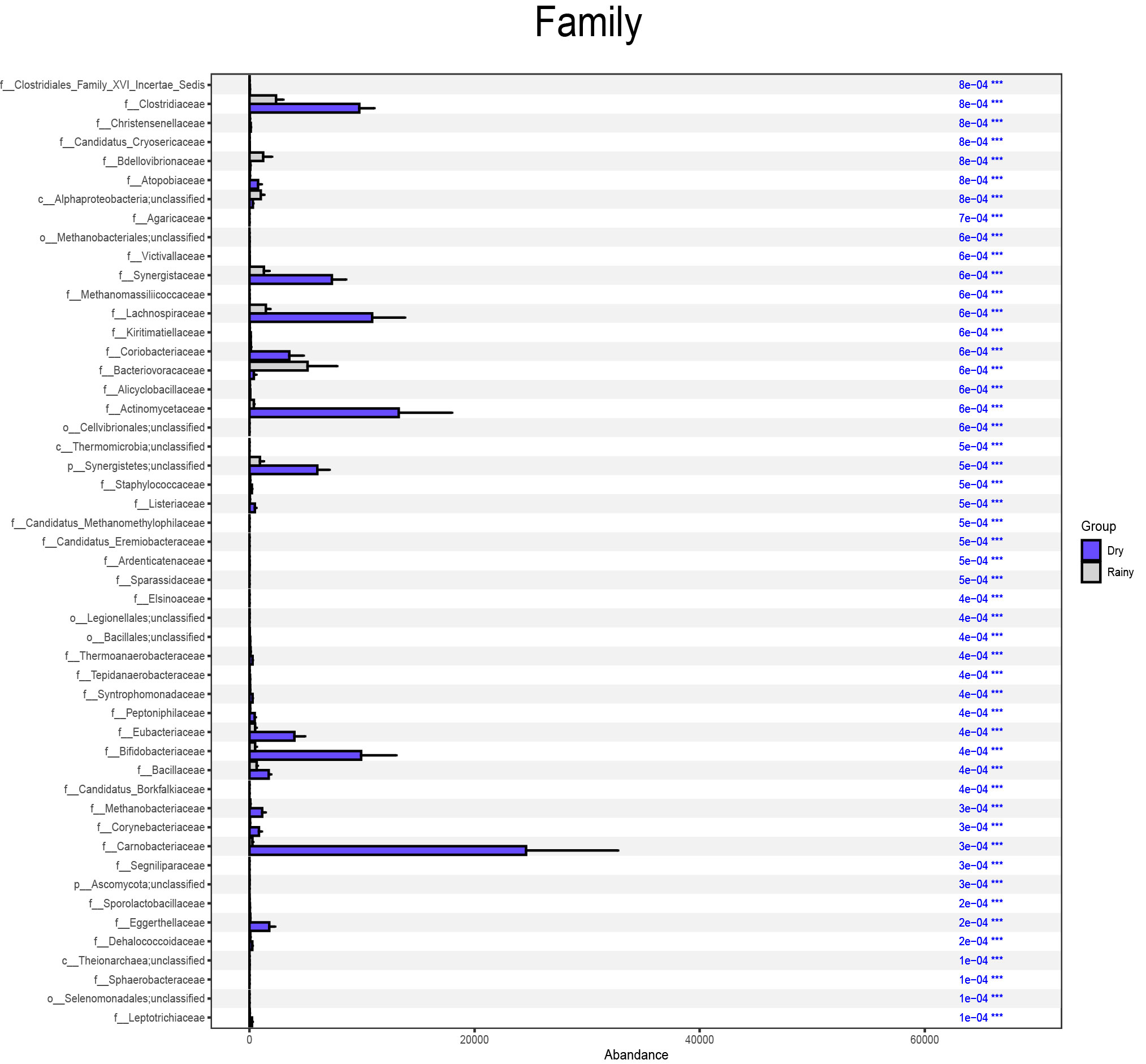

Supplement: Supplemental Information 2 [file peerj-13-20199-s002.jpg]

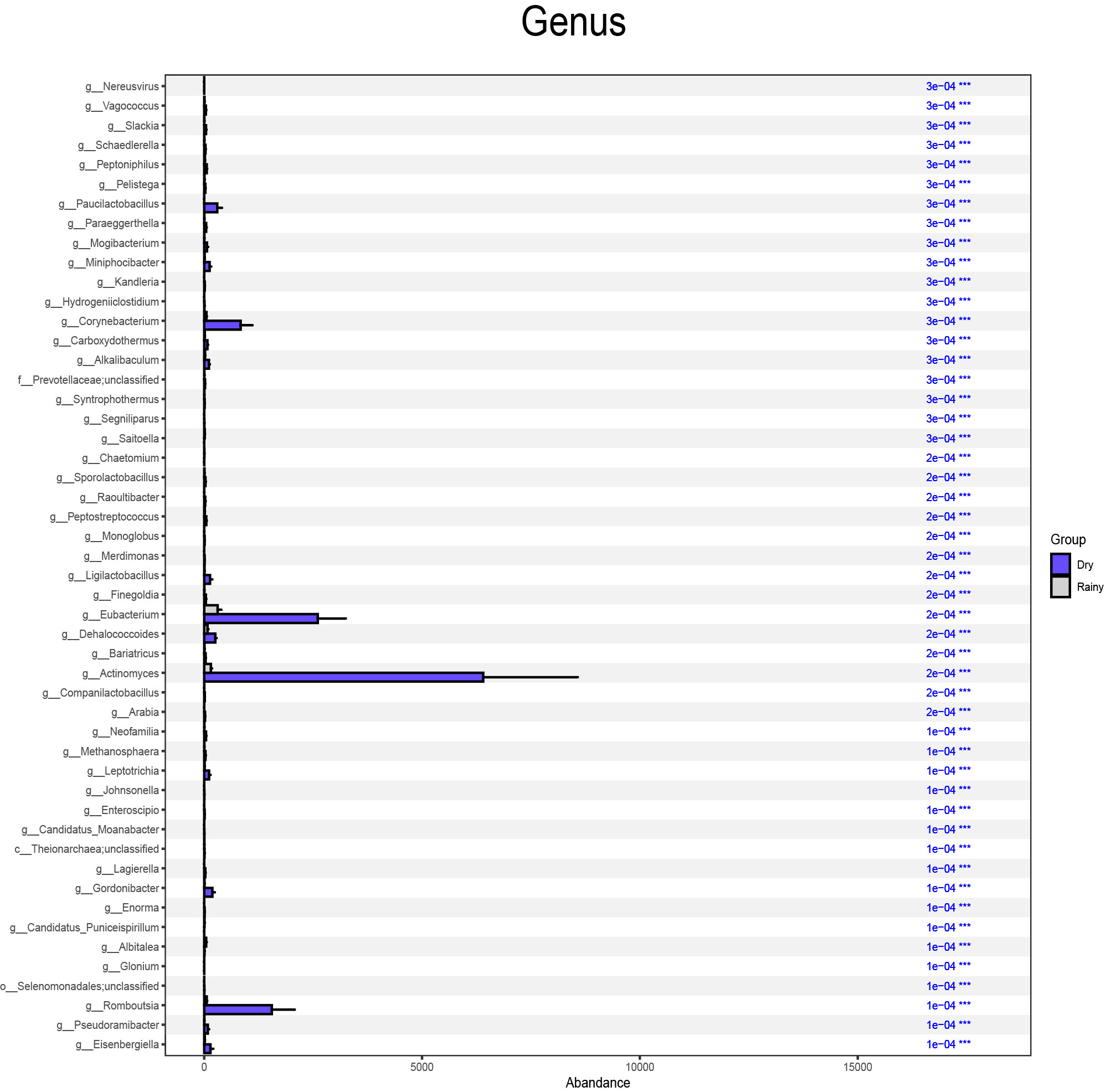

Supplement: Supplemental Information 3 [file peerj-13-20199-s003.jpg]

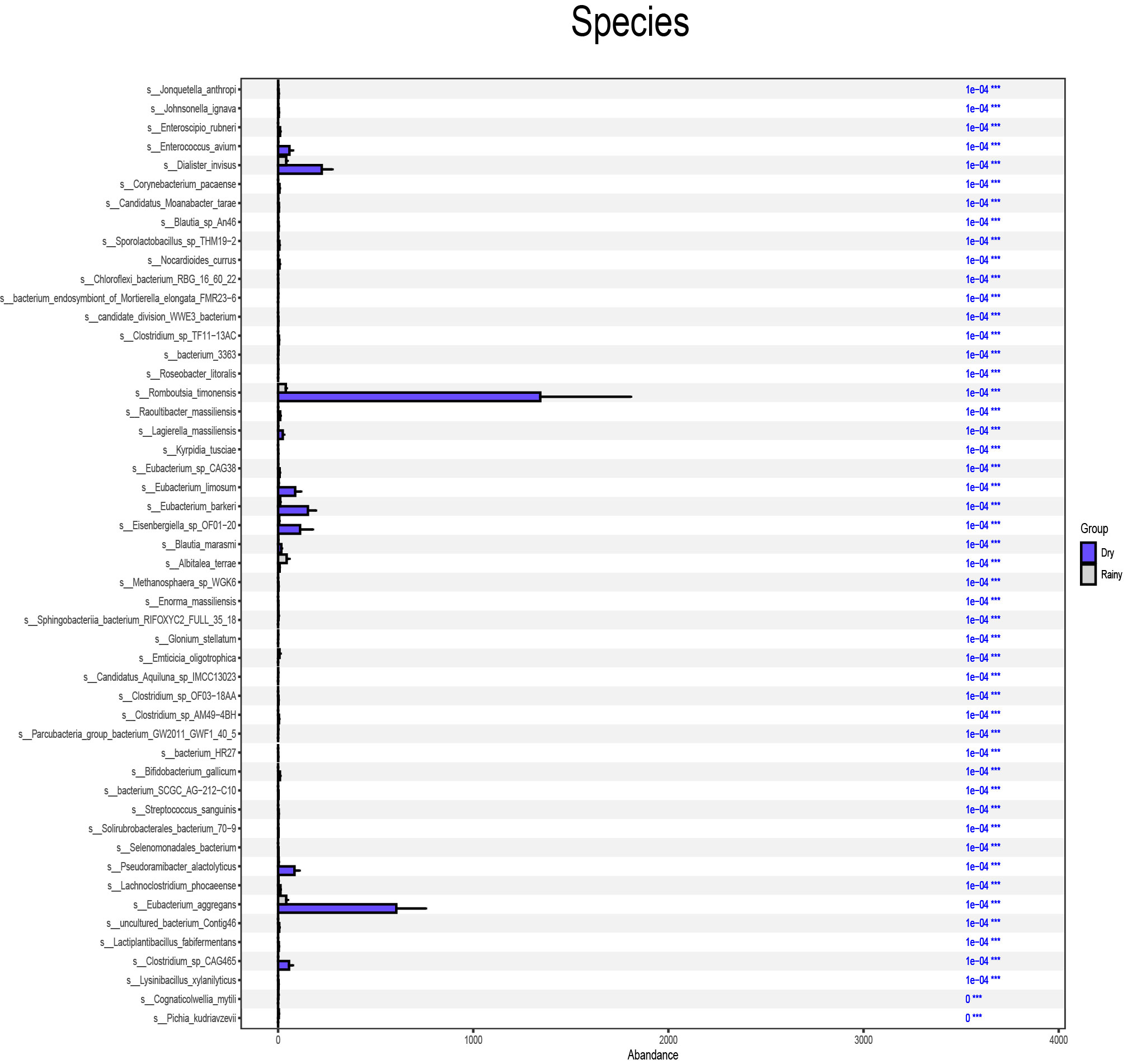

Supplement: Supplemental Information 4 [file peerj-13-20199-s004.jpg]

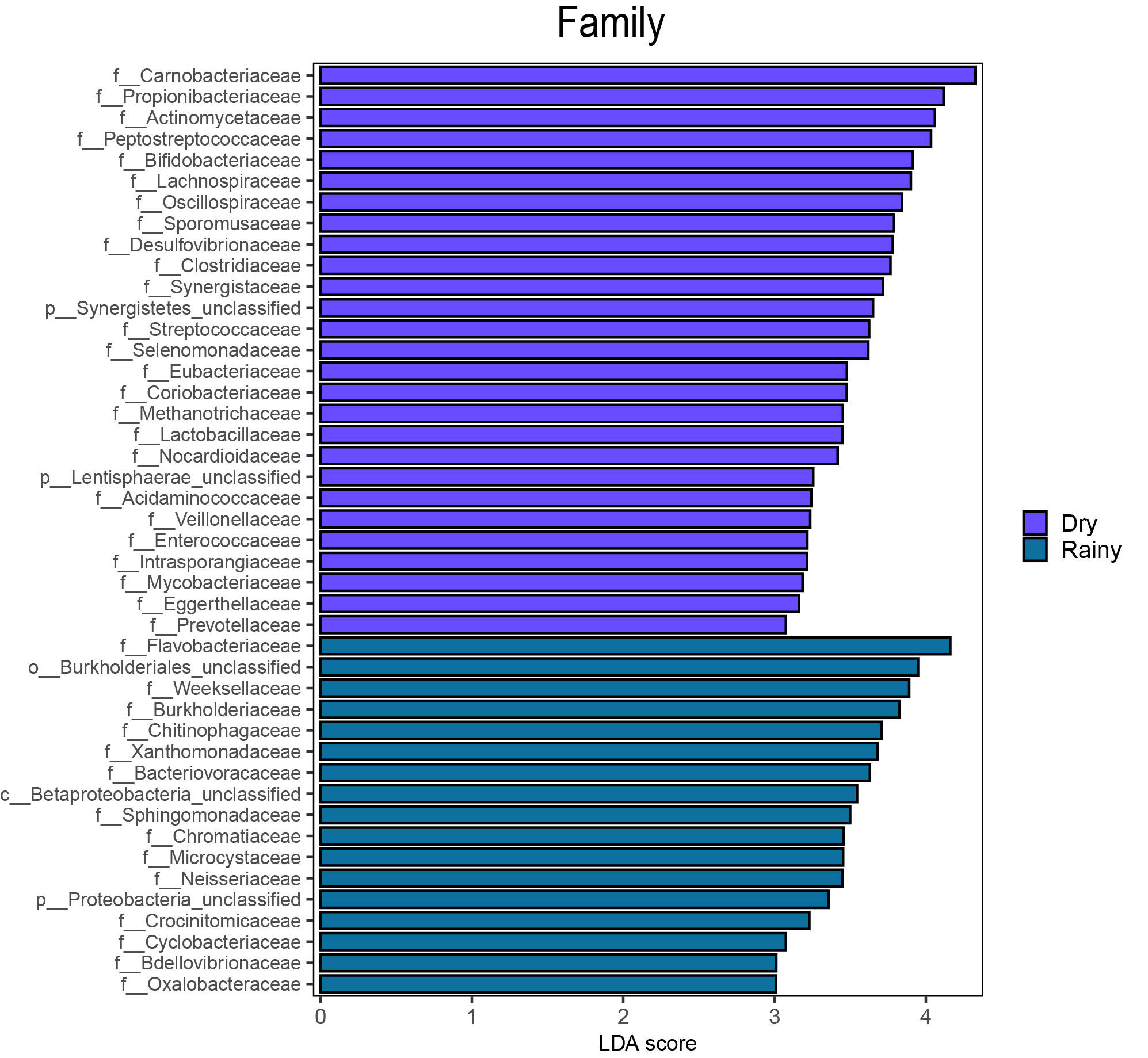

Supplement: Supplemental Information 5 [file peerj-13-20199-s005.jpg]

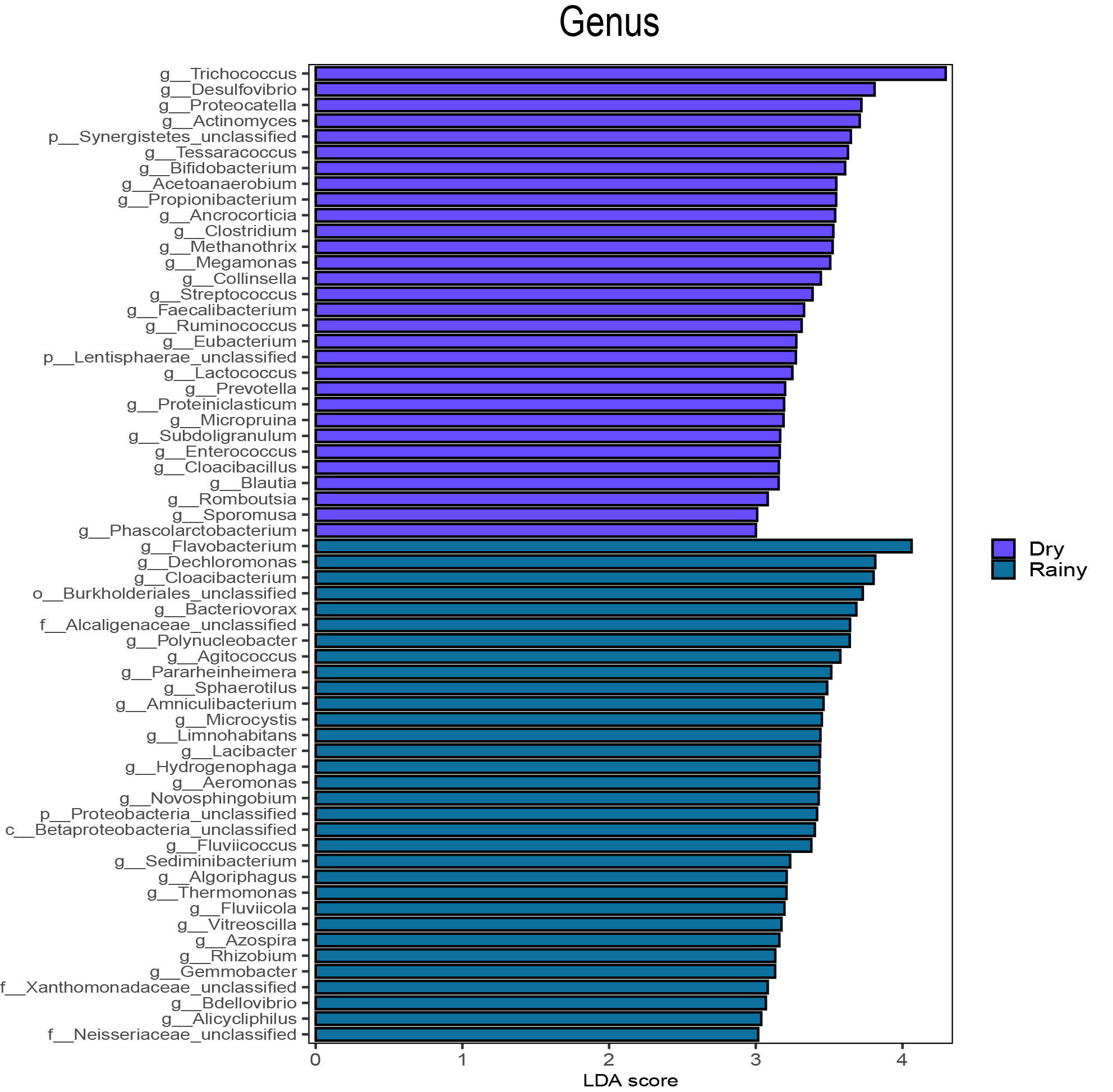

Supplement: Supplemental Information 6 [file peerj-13-20199-s006.jpg]

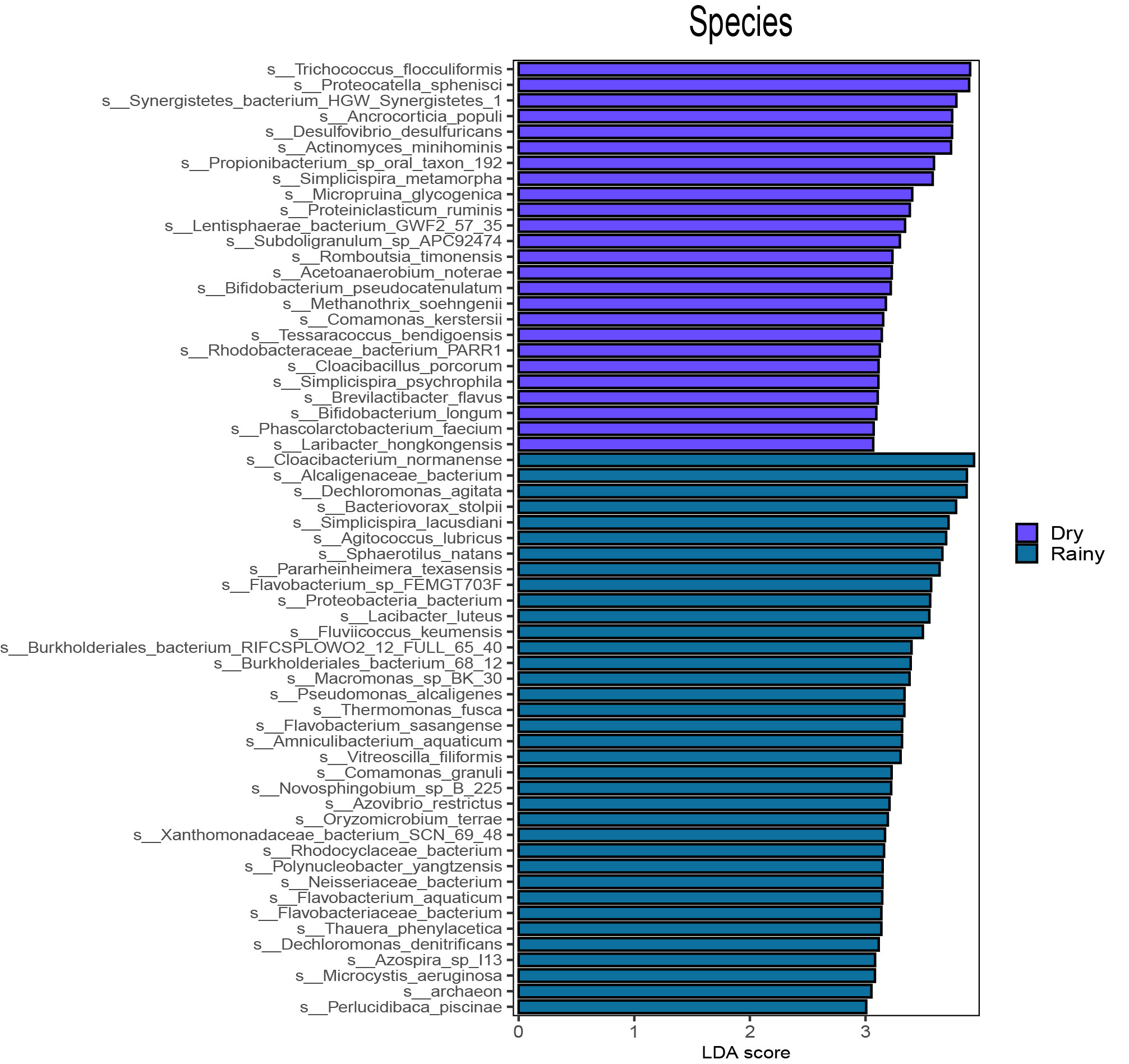

Supplement: Supplemental Information 7 [file peerj-13-20199-s007.jpg]

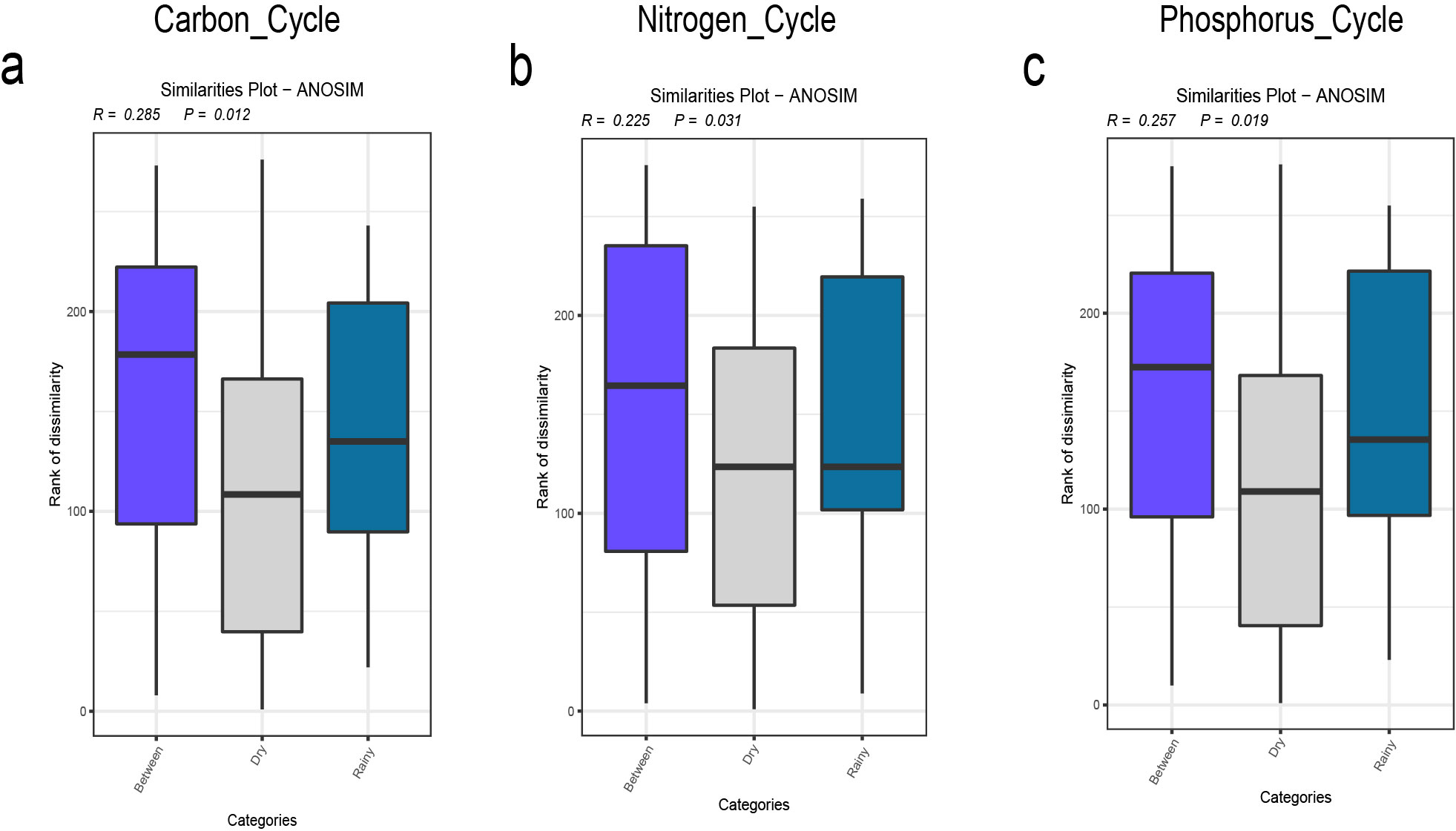

Supplement: Supplemental Information 8 [file peerj-13-20199-s008.jpg]
