# Supplementary material for: Effect of rainfall on metagenomics in a sewage environment in Hongta District, Yuxi city, Yunnan Province
Source: PeerJ. 2025 Nov 19;13:e20199. doi: 10.7717/peerj.20199 (PMC12640135; doi:10.7717/peerj.20199)
Supplement: Supplemental Information 9 [file peerj-13-20199-s009.docx]

**Metagenomic analysis workflow code**

#!/bin/bash

*# 宏基因组完整分析流程*

*# 包含: FASTP -> MEGAHIT -> MetaGeneMark -> Bowtie2 -> Diamond*

*# 设置参数*

PROJECT_NAME="my_metagenome_project"

RAW_DATA_DIR="raw_data"

CLEAN_DATA_DIR="cleaned_data"

ASSEMBLY_DIR="assembly"

GENE_PREDICTION_DIR="gene_prediction"

MAPPING_DIR="mapping"

ANNOTATION_DIR="annotation"

THREADS=32

MEMORY_LIMIT="0.9" *# MEGAHIT内存使用限制*

*# 数据库路径*

DIAMOND_NR_DB="/path/to/nr.dmnd"

DIAMOND_KEGG_DB="/path/to/kegg.dmnd"

DIAMOND_EGGNOG_DB="/path/to/eggnog.dmnd"

DIAMOND_CAZY_DB="/path/to/cazy.dmnd"

DIAMOND_PHI_DB="/path/to/phi.dmnd"

DIAMOND_VFDB_DB="/path/to/vfdb.dmnd"

DIAMOND_CARD_DB="/path/to/card.dmnd"

*# 创建目录结构*

mkdir -p $CLEAN_DATA_DIR $ASSEMBLY_DIR $GENE_PREDICTION_DIR $MAPPING_DIR $ANNOTATION_DIR

mkdir -p $ANNOTATION_DIR/nr $ANNOTATION_DIR/kegg $ANNOTATION_DIR/eggnog

mkdir -p $ANNOTATION_DIR/cazy $ANNOTATION_DIR/phi $ANNOTATION_DIR/vfdb $ANNOTATION_DIR/card

*# 记录开始时间*

echo "开始宏基因组分析流程: $(date)"

START_TIME=$(date +%s)

*# 获取样本列表*

SAMPLES=()

for R1_FILE in ${RAW_DATA_DIR}/*_R1.fastq.gz; do

SAMPLE_NAME=$(basename $R1_FILE | cut -d'_' -f1)

SAMPLES+=($SAMPLE_NAME)

done

*# 去重样本列表*

UNIQUE_SAMPLES=($(echo "${SAMPLES[@]}" | tr ' ' '\n' | sort -u | tr '\n' ' '))

echo "找到 ${#UNIQUE_SAMPLES[@]} 个样本: ${UNIQUE_SAMPLES[*]}"

*# 步骤1: FASTP质量控制*

echo "步骤1: FASTP质量控制..."

for SAMPLE in "${UNIQUE_SAMPLES[@]}"; do

echo "处理样本: $SAMPLE"

R1="${RAW_DATA_DIR}/${SAMPLE}_R1.fastq.gz"

R2="${RAW_DATA_DIR}/${SAMPLE}_R2.fastq.gz"

if [[ ! -f $R1 || ! -f $R2 ]]; then

echo "错误: 找不到样本 $SAMPLE 的读段文件"

continue

fi

*# 运行FASTP*

fastp --in1 $R1 --in2 $R2 \

--out1 $CLEAN_DATA_DIR/${SAMPLE}_R1_clean.fastq.gz \

--out2 $CLEAN_DATA_DIR/${SAMPLE}_R2_clean.fastq.gz \

--html $CLEAN_DATA_DIR/${SAMPLE}_fastp_report.html \

--json $CLEAN_DATA_DIR/${SAMPLE}_fastp_report.json \

--thread $THREADS \

--detect_adapter_for_pe \

--overrepresentation_analysis \

--correction \

--trim_poly_g \

--trim_poly_x \

--qualified_quality_phred 20 \

--unqualified_percent_limit 40 \

--length_required 50

echo "样本 $SAMPLE 质量控制完成"

done

*# 步骤2: MEGAHIT组装*

echo "步骤2: MEGAHIT组装..."

for SAMPLE in "${UNIQUE_SAMPLES[@]}"; do

echo "组装样本: $SAMPLE"

R1="$CLEAN_DATA_DIR/${SAMPLE}_R1_clean.fastq.gz"

R2="$CLEAN_DATA_DIR/${SAMPLE}_R2_clean.fastq.gz"

SAMPLE_ASSEMBLY_DIR="$ASSEMBLY_DIR/$SAMPLE"

*# 运行MEGAHIT*

megahit -1 $R1 -2 $R2 \

-o $SAMPLE_ASSEMBLY_DIR \

--k-list 31,51,71,91,111 \

--min-contig-len 500 \

-m $MEMORY_LIMIT \

-t $THREADS

*# 重命名contigs文件以包含样本名*

mv $SAMPLE_ASSEMBLY_DIR/final.contigs.fa $SAMPLE_ASSEMBLY_DIR/${SAMPLE}_contigs.fa

echo "样本 $SAMPLE 组装完成"

done

*# 步骤3: MetaGeneMark基因预测*

echo "步骤3: MetaGeneMark基因预测..."

for SAMPLE in "${UNIQUE_SAMPLES[@]}"; do

echo "预测样本: $SAMPLE 的基因"

CONTIGS_FILE="$ASSEMBLY_DIR/$SAMPLE/${SAMPLE}_contigs.fa"

SAMPLE_GENE_DIR="$GENE_PREDICTION_DIR/$SAMPLE"

mkdir -p $SAMPLE_GENE_DIR

*# 运行MetaGeneMark*

gmhmmp -m /path/to/MetaGeneMark_v1.mod \

-o $SAMPLE_GENE_DIR/${SAMPLE}.gff \

-f gff \

-a $SAMPLE_GENE_DIR/${SAMPLE}.faa \

-d $SAMPLE_GENE_DIR/${SAMPLE}.fna \

-s $SAMPLE_GENE_DIR/${SAMPLE}.stats \

$CONTIGS_FILE

echo "样本 $SAMPLE 基因预测完成"

done

*# 步骤4: Bowtie2读段比对*

echo "步骤4: Bowtie2读段比对..."

for SAMPLE in "${UNIQUE_SAMPLES[@]}"; do

echo "比对样本: $SAMPLE"

R1="$CLEAN_DATA_DIR/${SAMPLE}_R1_clean.fastq.gz"

R2="$CLEAN_DATA_DIR/${SAMPLE}_R2_clean.fastq.gz"

CONTIGS_FILE="$ASSEMBLY_DIR/$SAMPLE/${SAMPLE}_contigs.fa"

SAMPLE_MAPPING_DIR="$MAPPING_DIR/$SAMPLE"

mkdir -p $SAMPLE_MAPPING_DIR

*# 为每个样本的contigs构建Bowtie2索引*

bowtie2-build $CONTIGS_FILE $SAMPLE_MAPPING_DIR/${SAMPLE}_contigs_index

*# 运行Bowtie2比对*

bowtie2 -x $SAMPLE_MAPPING_DIR/${SAMPLE}_contigs_index \

-1 $R1 -2 $R2 \

-S $SAMPLE_MAPPING_DIR/${SAMPLE}.sam \

--threads $THREADS \

--sensitive \

--no-unal \

--un-conc-gz $SAMPLE_MAPPING_DIR/${SAMPLE}_unaligned_%.fq.gz

*# 转换SAM为BAM并排序*

samtools view -bS $SAMPLE_MAPPING_DIR/${SAMPLE}.sam | \

samtools sort -@ $THREADS -o $SAMPLE_MAPPING_DIR/${SAMPLE}_sorted.bam

*# 创建BAM索引*

samtools index $SAMPLE_MAPPING_DIR/${SAMPLE}_sorted.bam

*# 生成比对统计*

samtools flagstat $SAMPLE_MAPPING_DIR/${SAMPLE}_sorted.bam > $SAMPLE_MAPPING_DIR/${SAMPLE}_flagstat.txt

echo "样本 $SAMPLE 比对完成"

done

*# 步骤5: Diamond功能注释*

echo "步骤5: Diamond功能注释..."

*# 合并所有样本的基因序列*

cat $GENE_PREDICTION_DIR/*/*.faa > $ANNOTATION_DIR/all_samples_genes.faa

*# 对Nr数据库注释*

echo "注释Nr数据库..."

diamond blastp --query $ANNOTATION_DIR/all_samples_genes.faa \

--db $DIAMOND_NR_DB \

--out $ANNOTATION_DIR/nr/nr_annotations.tsv \

--outfmt 6 qseqid sseqid pident length mismatch gapopen qstart qend sstart send evalue bitscore stitle \

--evalue 1e-5 \

--max-target-seqs 5 \

--threads $THREADS \

--id 20 \

--query-cover 60

*# 对KEGG数据库注释*

echo "注释KEGG数据库..."

diamond blastp --query $ANNOTATION_DIR/all_samples_genes.faa \

--db $DIAMOND_KEGG_DB \

--out $ANNOTATION_DIR/kegg/kegg_annotations.tsv \

--outfmt 6 \

--evalue 1e-5 \

--max-target-seqs 1 \

--threads $THREADS \

--id 30 \

--query-cover 70

*# 对eggNOG数据库注释*

echo "注释eggNOG数据库..."

diamond blastp --query $ANNOTATION_DIR/all_samples_genes.faa \

--db $DIAMOND_EGGNOG_DB \

--out $ANNOTATION_DIR/eggnog/eggnog_annotations.tsv \

--outfmt 6 \

--evalue 1e-5 \

--max-target-seqs 1 \

--threads $THREADS \

--id 20 \

--query-cover 60

*# 对CAZy数据库注释*

echo "注释CAZy数据库..."

diamond blastp --query $ANNOTATION_DIR/all_samples_genes.faa \

--db $DIAMOND_CAZY_DB \

--out $ANNOTATION_DIR/cazy/cazy_annotations.tsv \

--outfmt 6 \

--evalue 1e-5 \

--max-target-seqs 1 \

--threads $THREADS \

--id 30 \

--query-cover 70

*# 对PHI数据库注释*

echo "注释PHI数据库..."

diamond blastp --query $ANNOTATION_DIR/all_samples_genes.faa \

--db $DIAMOND_PHI_DB \

--out $ANNOTATION_DIR/phi/phi_annotations.tsv \

--outfmt 6 \

--evalue 1e-5 \

--max-target-seqs 1 \

--threads $THREADS \

--id 40 \

--query-cover 80

*# 对VFDB数据库注释*

echo "注释VFDB数据库..."

diamond blastp --query $ANNOTATION_DIR/all_samples_genes.faa \

--db $DIAMOND_VFDB_DB \

--out $ANNOTATION_DIR/vfdb/vfdb_annotations.tsv \

--outfmt 6 \

--evalue 1e-5 \

--max-target-seqs 1 \

--threads $THREADS \

--id 40 \

--query-cover 80

*# 对CARD数据库注释*

echo "注释CARD数据库..."

diamond blastp --query $ANNOTATION_DIR/all_samples_genes.faa \

--db $DIAMOND_CARD_DB \

--out $ANNOTATION_DIR/card/card_annotations.tsv \

--outfmt 6 \

--evalue 1e-5 \

--max-target-seqs 1 \

--threads $THREADS \

--id 40 \

--query-cover 80

*# 步骤6: 生成结果摘要报告*

echo "步骤6: 生成结果摘要报告..."

SUMMARY_FILE="$PROJECT_NAME_analysis_summary.txt"

echo "宏基因组分析结果摘要" > $SUMMARY_FILE

echo "生成时间: $(date)" >> $SUMMARY_FILE

echo "项目名称: $PROJECT_NAME" >> $SUMMARY_FILE

echo "样本数量: ${#UNIQUE_SAMPLES[@]}" >> $SUMMARY_FILE

echo "样本列表: ${UNIQUE_SAMPLES[*]}" >> $SUMMARY_FILE

echo "" >> $SUMMARY_FILE

*# 添加每个样本的统计信息*

echo "各样本统计信息:" >> $SUMMARY_FILE

echo "==========================================" >> $SUMMARY_FILE

for SAMPLE in "${UNIQUE_SAMPLES[@]}"; do

*# 获取原始读段数量*

RAW_READS=$(zcat ${RAW_DATA_DIR}/${SAMPLE}_R1.fastq.gz | echo $((`wc -l`/4)))

*# 获取清洗后读段数量*

CLEAN_READS=$(zcat ${CLEAN_DATA_DIR}/${SAMPLE}_R1_clean.fastq.gz | echo $((`wc -l`/4)))

*# 获取contig数量和N50*

CONTIG_COUNT=$(grep -c ">" ${ASSEMBLY_DIR}/${SAMPLE}/${SAMPLE}_contigs.fa)

N50=$(awk '{if($0~/^>/){if(size>0){print size} size=0}else{size+=length($0)}}END{print size}' ${ASSEMBLY_DIR}/${SAMPLE}/${SAMPLE}_contigs.fa | sort -n | awk '{a[i++]=$1} END {print a[int(i*0.5)]}')

*# 获取预测基因数量*

GENE_COUNT=$(grep -c ">" ${GENE_PREDICTION_DIR}/${SAMPLE}/${SAMPLE}.faa)

*# 获取比对率*

MAPPING_RATE=$(grep "percentage of mapped reads" ${MAPPING_DIR}/${SAMPLE}/${SAMPLE}_flagstat.txt | awk '{print $1}')

*# 写入摘要*

echo "样本: $SAMPLE" >> $SUMMARY_FILE

echo " 原始读段数: $RAW_READS" >> $SUMMARY_FILE

echo " 清洗后读段数: $CLEAN_READS" >> $SUMMARY_FILE

echo " Contig数量: $CONTIG_COUNT" >> $SUMMARY_FILE

echo " N50: $N50" >> $SUMMARY_FILE

echo " 预测基因数: $GENE_COUNT" >> $SUMMARY_FILE

echo " 比对率: $MAPPING_RATE" >> $SUMMARY_FILE

echo "" >> $SUMMARY_FILE

done

*# 添加注释统计*

echo "功能注释统计:" >> $SUMMARY_FILE

echo "==========================================" >> $SUMMARY_FILE

echo "Nr注释基因数: $(wc -l < $ANNOTATION_DIR/nr/nr_annotations.tsv)" >> $SUMMARY_FILE

echo "KEGG注释基因数: $(wc -l < $ANNOTATION_DIR/kegg/kegg_annotations.tsv)" >> $SUMMARY_FILE

echo "eggNOG注释基因数: $(wc -l < $ANNOTATION_DIR/eggnog/eggnog_annotations.tsv)" >> $SUMMARY_FILE

echo "CAZy注释基因数: $(wc -l < $ANNOTATION_DIR/cazy/cazy_annotations.tsv)" >> $SUMMARY_FILE

echo "PHI注释基因数: $(wc -l < $ANNOTATION_DIR/phi/phi_annotations.tsv)" >> $SUMMARY_FILE

echo "VFDB注释基因数: $(wc -l < $ANNOTATION_DIR/vfdb/vfdb_annotations.tsv)" >> $SUMMARY_FILE

echo "CARD注释基因数: $(wc -l < $ANNOTATION_DIR/card/card_annotations.tsv)" >> $SUMMARY_FILE

*# 计算总运行时间*

END_TIME=$(date +%s)

ELAPSED_TIME=$(($END_TIME - $START_TIME))

echo "" >> $SUMMARY_FILE

echo "总运行时间: $(($ELAPSED_TIME / 3600))小时$((($ELAPSED_TIME % 3600) / 60))分钟$(($ELAPSED_TIME % 60))秒" >> $SUMMARY_FILE

echo "分析完成! 结果摘要保存在: $SUMMARY_FILE"

*# 步骤7: 生成可视化报告 (可选)*

echo "步骤7: 生成可视化报告..."

*# 这里可以添加生成图表和可视化报告的代码*

*# 例如使用R或Python生成统计图表*

echo "宏基因组分析流程全部完成!"

**R package data visualization and statistical analysis code**

r

*# 安装必要的R包*

if (!require("tidyverse")) install.packages("tidyverse")

if (!require("vegan")) install.packages("vegan")

if (!require("ggplot2")) install.packages("ggplot2")

if (!require("ggpubr")) install.packages("ggpubr")

if (!require("reshape2")) install.packages("reshape2")

if (!require("RColorBrewer")) install.packages("RColorBrewer")

if (!require("pheatmap")) install.packages("pheatmap")

if (!require("corrplot")) install.packages("corrplot")

if (!require("DESeq2")) {

if (!require("BiocManager")) install.packages("BiocManager")

BiocManager::install("DESeq2")

}

*# 加载包*

library(tidyverse)

library(vegan)

library(ggplot2)

library(ggpubr)

library(reshape2)

library(RColorBrewer)

library(pheatmap)

library(corrplot)

library(DESeq2)

*# 设置主题和颜色*

theme_set(theme_bw(base_size = 14))

my_colors <- c("#E69F00", "#56B4E9", "#009E73", "#F0E442", "#0072B2", "#D55E00", "#CC79A7")

*# 设置随机种子以确保结果可重现*

set.seed(123)

数据加载与预处理

*# 读取数据*

*# 物种丰度表（行是物种，列是样本）*

species_abundance <- read.csv("species_abundance.csv", row.names = 1, header = TRUE, check.names = FALSE)

*# 功能丰度表（如KEGG、COG等）*

function_abundance <- read.csv("function_abundance.csv", row.names = 1, header = TRUE, check.names = FALSE)

*# 样本元数据*

metadata <- read.csv("metadata.csv", row.names = 1, header = TRUE)

*# 确保数据一致性*

species_abundance <- species_abundance[, rownames(metadata)]

function_abundance <- function_abundance[, rownames(metadata)]

*# 数据预处理：过滤低丰度物种/功能*

filter_low_abundance <- function(abundance_table, threshold = 0.001) {

*# 计算每个物种/功能的平均相对丰度*

mean_abundance <- apply(abundance_table, 1, mean)

*# 保留平均相对丰度大于阈值的行*

filtered_table <- abundance_table[mean_abundance > threshold, ]

return(filtered_table)

}

species_filtered <- filter_low_abundance(species_abundance)

function_filtered <- filter_low_abundance(function_abundance)

*# 数据标准化（相对丰度）*

normalize_relative <- function(abundance_table) {

relative_abundance <- apply(abundance_table, 2, function(x) x/sum(x))

return(as.data.frame(t(relative_abundance)))

}

species_relative <- normalize_relative(species_filtered)

function_relative <- normalize_relative(function_filtered)

物种组成分析可视化

*# 1. 物种组成堆叠柱状图*

plot_taxa_barplot <- function(abundance_data, metadata, top_n = 10, group_var = "Group") {

*# 计算每个物种的平均丰度并选择前top_n个*

mean_abundance <- apply(abundance_data, 2, mean)

top_taxa <- names(sort(mean_abundance, decreasing = TRUE))[1:top_n]

*# 提取前top_n个物种的数据*

top_data <- abundance_data[, top_taxa]

*# 添加其他物种合并为"Others"*

top_data$Others <- 1 - rowSums(top_data)

*# 添加分组信息*

top_data$Sample <- rownames(top_data)

top_data <- merge(top_data, metadata, by.x = "Sample", by.y = "row.names")

*# 转换数据为长格式*

top_data_long <- melt(top_data, id.vars = c("Sample", group_var),

variable.name = "Taxa", value.name = "Abundance")

*# 绘制堆叠柱状图*

p <- ggplot(top_data_long, aes_string(x = "Sample", y = "Abundance", fill = "Taxa")) +

geom_bar(stat = "identity", position = "stack") +

facet_grid(as.formula(paste(".~", group_var)), scales = "free_x", space = "free") +

labs(x = "Sample", y = "Relative Abundance", title = "Top Taxa Composition") +

scale_fill_manual(values = colorRampPalette(brewer.pal(12, "Paired"))(top_n + 1)) +

theme(axis.text.x = element_text(angle = 45, hjust = 1),

legend.position = "right")

return(p)

}

*# 绘制物种组成图*

taxa_barplot <- plot_taxa_barplot(species_relative, metadata, top_n = 10, group_var = "Group")

print(taxa_barplot)

*# 2. 分组物种组成箱线图*

plot_taxa_boxplot <- function(abundance_data, metadata, taxa, group_var = "Group") {

*# 提取特定物种的数据*

plot_data <- data.frame(

Abundance = abundance_data[, taxa],

Sample = rownames(abundance_data)

)

*# 添加分组信息*

plot_data <- merge(plot_data, metadata, by.x = "Sample", by.y = "row.names")

*# 绘制箱线图*

p <- ggplot(plot_data, aes_string(x = group_var, y = "Abundance", fill = group_var)) +

geom_boxplot(alpha = 0.7) +

geom_jitter(position = position_jitter(width = 0.2), size = 2, alpha = 0.7) +

labs(x = group_var, y = "Relative Abundance", title = paste("Abundance of", taxa)) +

stat_compare_means(method = "kruskal.test", label = "p.format") +

scale_fill_manual(values = my_colors) +

theme(axis.text.x = element_text(angle = 45, hjust = 1))

return(p)

}

*# 绘制特定物种的箱线图*

taxa_boxplot <- plot_taxa_boxplot(species_relative, metadata, "Bacteroides", "Group")

print(taxa_boxplot)

功能组成分析可视化

*# 1. 功能通路丰度热图*

plot_function_heatmap <- function(abundance_data, metadata, top_n = 20, group_var = "Group") {

*# 计算每个功能的平均丰度并选择前top_n个*

mean_abundance <- apply(abundance_data, 2, mean)

top_functions <- names(sort(mean_abundance, decreasing = TRUE))[1:top_n]

*# 提取前top_n个功能的数据*

top_data <- abundance_data[, top_functions]

*# 添加分组信息用于注释*

annotation_df <- data.frame(Group = metadata[, group_var])

rownames(annotation_df) <- rownames(metadata)

*# 绘制热图*

pheatmap(t(as.matrix(top_data)),

scale = "row",

clustering_distance_rows = "euclidean",

clustering_distance_cols = "euclidean",

clustering_method = "complete",

annotation_col = annotation_df,

show_colnames = FALSE,

main = paste("Top", top_n, "Functional Pathways Heatmap"),

color = colorRampPalette(rev(brewer.pal(n = 7, name = "RdYlBu")))(100))

}

*# 绘制功能热图*

function_heatmap <- plot_function_heatmap(function_relative, metadata, top_n = 20, group_var = "Group")

*# 2. 特定功能通路在不同分组中的比较*

plot_function_comparison <- function(abundance_data, metadata, function_id, group_var = "Group") {

*# 提取特定功能的数据*

plot_data <- data.frame(

Abundance = abundance_data[, function_id],

Sample = rownames(abundance_data)

)

*# 添加分组信息*

plot_data <- merge(plot_data, metadata, by.x = "Sample", by.y = "row.names")

*# 绘制箱线图*

p <- ggplot(plot_data, aes_string(x = group_var, y = "Abundance", fill = group_var)) +

geom_boxplot(alpha = 0.7) +

geom_jitter(position = position_jitter(width = 0.2), size = 2, alpha = 0.7) +

labs(x = group_var, y = "Relative Abundance", title = paste("Abundance of", function_id)) +

stat_compare_means(method = "kruskal.test", label = "p.format") +

scale_fill_manual(values = my_colors) +

theme(axis.text.x = element_text(angle = 45, hjust = 1))

return(p)

}

*# 绘制特定功能通路的比较图*

function_comparison <- plot_function_comparison(function_relative, metadata, "K00001", "Group")

print(function_comparison)

Alpha多样性分析可视化

*# 计算Alpha多样性*

calculate_alpha_diversity <- function(abundance_data) {

*# 转换为矩阵*

data_matrix <- as.matrix(abundance_data)

*# 计算多样性指数*

alpha_df <- data.frame(

Sample = rownames(abundance_data),

Shannon = diversity(data_matrix, index = "shannon"),

Simpson = diversity(data_matrix, index = "simpson"),

Richness = apply(data_matrix > 0, 1, sum),

Evenness = diversity(data_matrix, index = "shannon") / log(apply(data_matrix > 0, 1, sum))

)

return(alpha_df)

}

*# 计算Alpha多样性*

alpha_diversity <- calculate_alpha_diversity(species_relative)

*# 添加分组信息*

alpha_diversity <- merge(alpha_diversity, metadata, by.x = "Sample", by.y = "row.names")

*# 可视化Alpha多样性*

plot_alpha_diversity <- function(alpha_df, group_var = "Group") {

*# 转换数据为长格式*

alpha_long <- melt(alpha_df, id.vars = c("Sample", group_var),

variable.name = "Index", value.name = "Value")

*# 绘制箱线图*

p <- ggplot(alpha_long, aes_string(x = group_var, y = "Value", fill = group_var)) +

geom_boxplot(alpha = 0.7) +

geom_jitter(position = position_jitter(width = 0.2), size = 2, alpha = 0.7) +

facet_wrap(~ Index, scales = "free_y") +

labs(x = group_var, y = "Value", title = "Alpha Diversity Indices") +

stat_compare_means(method = "kruskal.test", label = "p.format") +

scale_fill_manual(values = my_colors) +

theme(axis.text.x = element_text(angle = 45, hjust = 1))

return(p)

}

*# 绘制Alpha多样性图*

alpha_plot <- plot_alpha_diversity(alpha_diversity, "Group")

print(alpha_plot)

Beta多样性分析可视化

*# 计算Beta多样性*

calculate_beta_diversity <- function(abundance_data, method = "bray") {

*# 计算距离矩阵*

dist_matrix <- vegdist(abundance_data, method = method)

return(dist_matrix)

}

*# 执行PCoA分析*

perform_pcoa <- function(dist_matrix) {

pcoa <- cmdscale(dist_matrix, k = 2, eig = TRUE)

variance_exp <- pcoa$eig / sum(pcoa$eig) * 100

pcoa_df <- data.frame(

Sample = rownames(pcoa$points),

PCo1 = pcoa$points[, 1],

PCo2 = pcoa$points[, 2]

)

return(list(pcoa_df = pcoa_df, variance_exp = variance_exp))

}

*# 计算Bray-Curtis距离*

bray_dist <- calculate_beta_diversity(species_relative)

*# 执行PCoA分析*

pcoa_result <- perform_pcoa(bray_dist)

pcoa_df <- pcoa_result$pcoa_df

variance_exp <- pcoa_result$variance_exp

*# 添加分组信息*

pcoa_df <- merge(pcoa_df, metadata, by.x = "Sample", by.y = "row.names")

*# 可视化PCoA结果*

plot_pcoa <- function(pcoa_df, variance_exp, group_var = "Group") {

p <- ggplot(pcoa_df, aes_string(x = "PCo1", y = "PCo2", color = group_var)) +

geom_point(size = 3, alpha = 0.7) +

stat_ellipse(level = 0.95, linetype = 2) +

labs(

x = paste0("PCo1 (", round(variance_exp[1], 2), "%)"),

y = paste0("PCo2 (", round(variance_exp[2], 2), "%)"),

title = "Beta Diversity PCoA Plot"

) +

scale_color_manual(values = my_colors) +

theme(legend.position = "right")

return(p)

}

*# 绘制PCoA图*

pcoa_plot <- plot_pcoa(pcoa_df, variance_exp, "Group")

print(pcoa_plot)

*# PERMANOVA分析（组间差异检验）*

perform_permanova <- function(dist_matrix, metadata, formula_str) {

permanova <- adonis2(

as.formula(formula_str),

data = metadata,

permutations = 999,

method = "bray"

)

return(permanova)

}

*# 执行PERMANOVA*

permanova_result <- perform_permanova(bray_dist, metadata, "bray_dist ~ Group")

print(permanova_result)

差异丰度分析可视化

*# 使用DESeq2进行差异丰度分析*

perform_deseq2 <- function(abundance_data, metadata, group_var = "Group", reference = NULL) {

*# 创建DESeq2对象*

dds <- DESeqDataSetFromMatrix(

countData = round(abundance_data * 10000), *# 将相对丰度转换为伪计数*

colData = metadata,

design = as.formula(paste("~", group_var))

)

*# 设置参考水平（如果有的话）*

if (!is.null(reference)) {

dds[[group_var]] <- relevel(dds[[group_var]], ref = reference)

}

*# 运行DESeq2分析*

dds <- DESeq(dds)

*# 提取结果*

res <- results(dds)

res_df <- as.data.frame(res)

res_df$Feature <- rownames(res_df)

return(res_df)

}

*# 执行差异丰度分析*

deseq_results <- perform_deseq2(species_filtered, metadata, "Group", reference = "Control")

*# 可视化差异分析结果*

plot_volcano <- function(deseq_results, p_cutoff = 0.05, fc_cutoff = 2) {

*# 准备数据*

volcano_data <- deseq_results %>%

mutate(

Significance = case_when(

padj < p_cutoff & log2FoldChange > log2(fc_cutoff) ~ "Up",

padj < p_cutoff & log2FoldChange < -log2(fc_cutoff) ~ "Down",

TRUE ~ "Not significant"

)

)

*# 绘制火山图*

p <- ggplot(volcano_data, aes(x = log2FoldChange, y = -log10(padj), color = Significance)) +

geom_point(alpha = 0.7, size = 2) +

scale_color_manual(values = c("Down" = "blue", "Not significant" = "gray", "Up" = "red")) +

geom_hline(yintercept = -log10(p_cutoff), linetype = "dashed", color = "black") +

geom_vline(xintercept = c(-log2(fc_cutoff), log2(fc_cutoff)), linetype = "dashed", color = "black") +

labs(x = "log2(Fold Change)", y = "-log10(Adjusted p-value)", title = "Volcano Plot") +

theme(legend.position = "right")

return(p)

}

*# 绘制火山图*

volcano_plot <- plot_volcano(deseq_results)

print(volcano_plot)

*# 绘制差异物种热图*

plot_differential_heatmap <- function(abundance_data, deseq_results, metadata,

p_cutoff = 0.05, fc_cutoff = 2, top_n = 20,

group_var = "Group") {

*# 筛选显著差异的物种*

sig_features <- deseq_results %>%

filter(padj < p_cutoff & abs(log2FoldChange) > log2(fc_cutoff)) %>%

arrange(padj) %>%

head(top_n) %>%

pull(Feature)

*# 提取显著差异物种的数据*

sig_data <- abundance_data[, sig_features, drop = FALSE]

*# 添加分组信息用于注释*

annotation_df <- data.frame(Group = metadata[, group_var])

rownames(annotation_df) <- rownames(metadata)

*# 绘制热图*

pheatmap(t(as.matrix(sig_data)),

scale = "row",

clustering_distance_rows = "euclidean",

clustering_distance_cols = "euclidean",

clustering_method = "complete",

annotation_col = annotation_df,

show_colnames = FALSE,

main = "Differentially Abundant Features Heatmap",

color = colorRampPalette(rev(brewer.pal(n = 7, name = "RdYlBu")))(100))

}

*# 绘制差异物种热图*

differential_heatmap <- plot_differential_heatmap(species_relative, deseq_results, metadata)

功能富集分析可视化

*# 功能富集分析（以KEGG通路为例）*

perform_enrichment <- function(deseq_results, pathway_annotation, p_cutoff = 0.05, fc_cutoff = 2) {

*# 筛选显著差异的物种*

sig_features <- deseq_results %>%

filter(padj < p_cutoff & abs(log2FoldChange) > log2(fc_cutoff)) %>%

pull(Feature)

*# 获取这些物种的功能注释*

sig_pathways <- pathway_annotation %>%

filter(Feature %in% sig_features) %>%

group_by(Pathway) %>%

summarize(Count = n()) %>%

arrange(desc(Count))

return(sig_pathways)

}

*# 假设我们有 pathway_annotation 数据框，包含物种到KEGG通路的映射*

*# pathway_annotation <- read.csv("pathway_annotation.csv", header = TRUE)*

*# 执行富集分析*

*# enrichment_results <- perform_enrichment(deseq_results, pathway_annotation)*

*# 可视化富集结果*

plot_enrichment <- function(enrichment_results, top_n = 15) {

*# 选择前top_n个富集通路*

top_pathways <- enrichment_results %>%

arrange(desc(Count)) %>%

head(top_n)

*# 绘制条形图*

p <- ggplot(top_pathways, aes(x = reorder(Pathway, Count), y = Count)) +

geom_bar(stat = "identity", fill = "steelblue", alpha = 0.7) +

coord_flip() +

labs(x = "Pathway", y = "Number of Features", title = "Pathway Enrichment Analysis") +

theme(axis.text.y = element_text(size = 10))

return(p)

}

*# 绘制富集分析图*

*# enrichment_plot <- plot_enrichment(enrichment_results)*

*# print(enrichment_plot)*

相关性分析可视化

*# 计算物种与环境因子的相关性*

calculate_correlation <- function(abundance_data, env_data, method = "spearman") {

*# 计算相关性矩阵*

cor_matrix <- cor(abundance_data, env_data, method = method)

return(cor_matrix)

}

*# 假设我们有环境因子数据*

*# env_data <- read.csv("environmental_factors.csv", row.names = 1, header = TRUE)*

*# 计算相关性*

*# cor_results <- calculate_correlation(species_relative, env_data)*

*# 可视化相关性热图*

plot_correlation_heatmap <- function(cor_matrix, p_matrix = NULL) {

*# 创建相关性热图*

corrplot(cor_matrix,

method = "color",

type = "full",

order = "hclust",

tl.cex = 0.7,

tl.col = "black",

col = colorRampPalette(rev(brewer.pal(n = 7, name = "RdYlBu")))(100),

main = "Correlation Heatmap")

*# 添加显著性标记（如果有p值矩阵）*

if (!is.null(p_matrix)) {

corrplot(cor_matrix,

p.mat = p_matrix,

insig = "label_sig",

sig.level = c(0.001, 0.01, 0.05),

pch.cex = 0.8,

pch.col = "white",

add = TRUE)

}

}

*# 绘制相关性热图*

*# plot_correlation_heatmap(cor_results)*

*# Mantel检验（物种组成与环境因子的相关性）*

perform_mantel_test <- function(species_data, env_data, method = "bray") {

*# 计算物种距离矩阵*

species_dist <- vegdist(species_data, method = method)

*# 计算环境距离矩阵*

env_dist <- vegdist(env_data, method = "euclidean")

*# 执行Mantel检验*

mantel_test <- mantel(species_dist, env_dist, method = "spearman", permutations = 999)

return(mantel_test)

}

*# 执行Mantel检验*

*# mantel_result <- perform_mantel_test(species_relative, env_data)*

*# print(mantel_result)*

保存结果和图形

*# 保存图形*

ggsave("taxa_composition.png", taxa_barplot, width = 12, height = 8, dpi = 300)

ggsave("alpha_diversity.png", alpha_plot, width = 10, height = 6, dpi = 300)

ggsave("beta_diversity_pcoa.png", pcoa_plot, width = 8, height = 6, dpi = 300)

ggsave("volcano_plot.png", volcano_plot, width = 8, height = 6, dpi = 300)

*# 保存统计结果*

write.csv(alpha_diversity, "alpha_diversity_results.csv", row.names = FALSE)

write.csv(deseq_results, "differential_abundance_results.csv", row.names = FALSE)

*# 保存PERMANOVA结果*

sink("permanova_results.txt")

print(permanova_result)

sink()

*# 保存会话信息（便于复现）*

sink("session_info.txt")

sessionInfo()

sink()

**Application of FDR correction in metagenomic analysis**

r

*# 模拟宏基因组物种丰度数据*

n_species <- 1000

n_samples <- 30

*# 生成物种丰度矩阵（使用负二项分布模拟计数数据）*

abundance_matrix <- matrix(

rnbinom(n_species * n_samples, mu = 500, size = 5),

nrow = n_species,

ncol = n_samples

)

rownames(abundance_matrix) <- paste0("species_", 1:n_species)

colnames(abundance_matrix) <- paste0("sample_", 1:n_samples)

*# 创建分组信息（例如疾病vs健康）*

group <- factor(rep(c("healthy", "disease"), each = n_samples/2))

*# 模拟一些真正差异丰度的物种*

da_species <- sample(1:n_species, 100) *# 100个差异丰度物种*

for (i in da_species) {

abundance_matrix[i, group == "disease"] <-

abundance_matrix[i, group == "disease"] * runif(1, 2, 10)

}

*# 执行Wilcoxon秩和检验（适用于微生物组数据）*

p_values <- apply(abundance_matrix, 1, function(x) {

wilcox.test(x ~ group)$p.value

})

*# 计算中位数折叠变化*

median_fc <- apply(abundance_matrix, 1, function(x) {

median_disease <- median(x[group == "disease"])

median_healthy <- median(x[group == "healthy"])

median_disease / median_healthy

})

*# 应用FDR校正*

qvalues <- p.adjust(p_values, method = "BH")

*# 创建结果数据框*

da_results <- data.frame(

species = rownames(abundance_matrix),

p_value = p_values,

fold_change = median_fc,

log2_fold_change = log2(median_fc),

qvalue = qvalues,

significant = qvalues < 0.05 *# FDR < 5%*

)

*# 查看显著差异丰度物种数量*

cat("显著差异丰度物种数量 (FDR < 0.05):", sum(da_results$significant), "\n")

*# 绘制曼哈顿图展示FDR校正结果*

manhattan_plot <- ggplot(da_results, aes(x = 1:nrow(da_results), y = -log10(p_value),

color = significant, size = abs(log2_fold_change))) +

geom_point(alpha = 0.7) +

scale_color_manual(values = c("gray", "red")) +

geom_hline(yintercept = -log10(max(da_results$p_value[da_results$significant])),

linetype = "dashed", color = "blue") +

labs(

x = "Species Index",

y = "-Log10 P-value",

title = "Manhattan Plot for Species Differential Abundance",

subtitle = paste0("Significant species (FDR < 0.05): ", sum(da_results$significant))

) +

theme_minimal() +

theme(legend.position = "none")

print(manhattan_plot)

*# 筛选并保存显著结果*

significant_species <- da_results %>%

filter(significant) %>%

arrange(qvalue)

write.csv(significant_species, "significant_species_fdr_0.05.csv", row.names = FALSE)

r

*# 模拟功能富集分析结果（如KEGG通路富集分析）*

n_pathways <- 500

*# 生成模拟通路富集结果*

pathway_results <- data.frame(

pathway_id = paste0("pathway_", 1:n_pathways),

pathway_name = paste("Pathway", 1:n_pathways),

p_value = runif(n_pathways, 0, 0.1), *# 模拟p值*

enrichment_score = runif(n_pathways, -2, 2) *# 模拟富集分数*

)

*# 随机选择一些通路作为真正富集的通路*

true_enriched <- sample(1:n_pathways, 50)

pathway_results$p_value[true_enriched] <- runif(50, 0, 0.01) *# 使这些通路的p值更小*

*# 应用FDR校正*

pathway_results$qvalue <- p.adjust(pathway_results$p_value, method = "BH")

pathway_results$significant <- pathway_results$qvalue < 0.05

*# 查看显著富集通路数量*

cat("显著富集通路数量 (FDR < 0.05):", sum(pathway_results$significant), "\n")

*# 绘制富集分析结果图*

enrichment_plot <- pathway_results %>%

arrange(p_value) %>%

mutate(log10p = -log10(p_value)) %>%

ggplot(aes(x = enrichment_score, y = log10p, color = significant, label = pathway_name)) +

geom_point(alpha = 0.7, size = 3) +

scale_color_manual(values = c("gray", "red")) +

geom_text_repel(

data = subset(pathway_results, significant & abs(enrichment_score) > 1),

size = 3,

max.overlaps = 15

) +

labs(

x = "Enrichment Score",

y = "-Log10 P-value",

title = "Pathway Enrichment Analysis with FDR Correction",

subtitle = paste0("Significant pathways (FDR < 0.05): ", sum(pathway_results$significant))

) +

theme_minimal() +

theme(legend.position = "none")

print(enrichment_plot)

*# 保存富集分析结果*

write.csv(pathway_results, "pathway_enrichment_results_with_fdr.csv", row.names = FALSE)
