# Supplementary material for: Effect of rainfall on metagenomics in a sewage environment in Hongta District, Yuxi city, Yunnan Province
Source: PeerJ. 2025 Nov 19;13:e20199. doi: 10.7717/peerj.20199 (PMC12640135; doi:10.7717/peerj.20199)
Supplement: Supplemental Information 11 [file peerj-13-20199-s011.docx]

Table S2. Data quality control results for all sequencing samples

| Sample name | Total Reads | Clean Reads | Percentage | Clean bases | GC Content | %>Q20 | %>Q30 |
| --- | --- | --- | --- | --- | --- | --- | --- |
| HT0101 | 70,818,004 | 70,330,228 | 99.31% | 10,535,199,241 | 54.18% | 97.90% | 93.92% |
| HT0108 | 75,040,368 | 74,505,148 | 99.29% | 11,161,560,546 | 50.12% | 97.73% | 93.45% |
| HT0201 | 74,855,168 | 74,278,860 | 99.23% | 11,124,658,174 | 53.29% | 97.65% | 93.34% |
| HT0208 | 73,612,084 | 73,121,132 | 99.33% | 10,956,211,312 | 49.78% | 97.77% | 93.55% |
| HT0301 | 69,847,304 | 69,394,590 | 99.35% | 10,391,613,126 | 56.57% | 97.86% | 93.87% |
| HT0308 | 75,399,500 | 74,772,670 | 99.17% | 11,195,355,424 | 52.68% | 97.76% | 93.58% |
| HT0401 | 70,094,000 | 69,090,180 | 98.57% | 9,633,699,735 | 49.89% | 98.35% | 94.95% |
| HT0408 | 67,256,652 | 66,512,560 | 98.89% | 9,349,273,049 | 47.91% | 98.08% | 94.44% |
| HT0501 | 75,452,076 | 74,415,966 | 98.63% | 10,948,459,734 | 37.91% | 98.13% | 94.25% |
| HT0508 | 107,677,338 | 106,719,360 | 99.11% | 15,387,491,335 | 50.45% | 98.28% | 94.87% |
| HT0601 | 80,547,930 | 79,495,232 | 98.69% | 11,606,711,454 | 41.03% | 98.06% | 94.12% |
| HT0608 | 106,874,480 | 105,795,210 | 98.99% | 15,204,449,978 | 46.88% | 98.30% | 94.86% |
| HT0701 | 77,755,524 | 76,938,150 | 98.95% | 11,135,916,948 | 46.93% | 98.15% | 94.49% |
| HT0708 | 94,339,386 | 92,802,876 | 98.37% | 13,184,231,598 | 46.07% | 98.32% | 94.95% |
| HT0801 | 78,546,786 | 77,800,350 | 99.05% | 11,105,540,062 | 51.45% | 98.35% | 95.02% |
| HT0808 | 103,835,318 | 102,629,536 | 98.84% | 14,759,619,632 | 52.13% | 98.26% | 94.92% |
| HT0901 | 75,653,466 | 74,851,030 | 98.94% | 10,753,449,994 | 39.05% | 98.19% | 94.42% |
| HT0908 | 88,998,224 | 88,082,200 | 98.97% | 12,768,479,106 | 45.45% | 98.30% | 94.82% |
| HT1001 | 78,494,304 | 77,756,788 | 99.06% | 11,304,703,441 | 48.12% | 98.15% | 94.46% |
| HT1008 | 84,234,648 | 83,352,786 | 98.95% | 11,978,861,955 | 42.80% | 98.29% | 94.76% |
| HT1101 | 72,557,042 | 72,007,108 | 99.24% | 10,638,545,543 | 47.64% | 98.24% | 94.70% |
| HT1108 | 109,248,326 | 108,029,794 | 98.88% | 15,228,767,812 | 50.31% | 98.33% | 95.04% |
| HT1201 | 61,126,818 | 60,628,586 | 99.18% | 8,913,049,298 | 50.08% | 98.09% | 94.42% |
| HT1208 | 101,371,498 | 100,012,442 | 98.66% | 14,253,359,993 | 51.36% | 98.24% | 94.87% |
