# Supplementary material for: Effect of rainfall on metagenomics in a sewage environment in Hongta District, Yuxi city, Yunnan Province
Source: PeerJ. 2025 Nov 19;13:e20199. doi: 10.7717/peerj.20199 (PMC12640135; doi:10.7717/peerj.20199)
Supplement: Supplemental Information 12 [file peerj-13-20199-s012.docx]

Table S3. Assembly statistics for the sequencing data

| sample | ORF_nums | num_contigs | total_length(bp) | min_length | max_length | average_length | N50 |
| --- | --- | --- | --- | --- | --- | --- | --- |
| HT0101 | 637545 | 349,785 | 404,739,402 | 500 | 107,542 | 1,157.10 | 1,255 |
| HT0201 | 654851 | 380,601 | 405,178,302 | 500 | 104,189 | 1,064.60 | 1,097 |
| HT0301 | 558865 | 313,477 | 351,982,146 | 500 | 165,599 | 1,122.80 | 1,182 |
| HT0401 | 385280 | 248,573 | 241,971,519 | 500 | 75,440 | 973.4 | 982 |
| HT0501 | 268708 | 253,237 | 228,523,353 | 500 | 124,097 | 902.4 | 877 |
| HT0601 | 361049 | 331,883 | 301,151,049 | 500 | 41,532 | 907.4 | 891 |
| HT0701 | 495762 | 370,529 | 343,665,725 | 500 | 314,437 | 927.5 | 903 |
| HT0801 | 485867 | 321,088 | 306,033,544 | 500 | 105,523 | 953.1 | 939 |
| HT0901 | 320406 | 280,482 | 284,949,793 | 500 | 155,057 | 1,015.90 | 1,057 |
| HT1001 | 533323 | 348,175 | 345,814,232 | 500 | 68,688 | 993.2 | 1,003 |
| HT1101 | 572114 | 402,200 | 407,164,944 | 500 | 86,991 | 1,012.30 | 1,050 |
| HT1201 | 514826 | 336,271 | 326,040,937 | 500 | 173,716 | 969.6 | 967 |
| HT0108 | 683937 | 372,703 | 434,860,194 | 500 | 142,965 | 1,166.80 | 1,280 |
| HT0208 | 669758 | 363,941 | 428,515,782 | 500 | 129,067 | 1,177.40 | 1,298 |
| HT0308 | 708505 | 390,010 | 451,899,251 | 500 | 189,997 | 1,158.70 | 1,264 |
| HT0408 | 433141 | 287,499 | 304,771,339 | 500 | 116,558 | 1,060.10 | 1,113 |
| HT0508 | 717551 | 413,813 | 441,654,706 | 500 | 127,063 | 1,067.30 | 1,119 |
| HT0608 | 560247 | 374,860 | 394,254,243 | 500 | 63,654 | 1,051.70 | 1,098 |
| HT0708 | 412932 | 311,103 | 304,782,164 | 500 | 58,641 | 979.7 | 992 |
| HT0808 | 625268 | 402,031 | 358,343,550 | 500 | 139,728 | 891.3 | 867 |
| HT0908 | 567272 | 349,705 | 353,641,208 | 500 | 137,508 | 1,011.30 | 1,036 |
| HT1008 | 376108 | 258,167 | 296,231,066 | 500 | 133,501 | 1,147.40 | 1,256 |
| HT1108 | 629561 | 370,203 | 374,455,668 | 500 | 83,363 | 1,011.50 | 1,037 |
| HT1208 | 556364 | 316,312 | 329,208,199 | 500 | 133,655 | 1,040.80 | 1,081 |
