# Supplementary material for: Effect of rainfall on metagenomics in a sewage environment in Hongta District, Yuxi city, Yunnan Province
Source: PeerJ. 2025 Nov 19;13:e20199. doi: 10.7717/peerj.20199 (PMC12640135; doi:10.7717/peerj.20199)
Supplement: Supplemental Information 13 [file peerj-13-20199-s013.docx]

# Metagenomic Analysis Workflow

# Includes

# Parameter Configuration

Memory Usage Limits

# Database Path

# Directory Structure Creation

# Start Time Logging

Initiate Metagenomic Analysis Workflow

# Retrieve Sample List

# Dedupe Sample List

Found

samples

# Step 1: FASTP Quality Control

echo ‘Step 1: FASTP quality control...’

echo ‘Processing sample: $SAMPLE’

echo ‘Error: Could not locate read files for sample $SAMPLE’

# Run FASTP

echo ‘Quality control completed for sample $SAMPLE’

Step 2: MEGAHIT Assembly

echo ‘Step 2: MEGAHIT Assembly...’

echo ‘Assembling sample: $SAMPLE’

# Run MEGAHIT

# Rename contig files to include sample name

echo ‘Assembly of sample $SAMPLE completed’

# Step 3: MetaGeneMark Gene Prediction

echo ‘Step 3: MetaGeneMark gene prediction...’

echo ‘Predicting genes for sample: $SAMPLE’

# Run MetaGeneMark

echo ‘Gene prediction for sample $SAMPLE completed’

# Step 4: Bowtie2 read alignment

echo ‘Step 4: Bowtie2 read alignment...’

echo ‘Matching sample: $SAMPLE’

# Build Bowtie2 indexes for each sample's contigs

# Run Bowtie2 alignment

# Convert SAM to BAM and sort

# Create BAM index

# Generate alignment statistics

echo ‘Alignment for sample $SAMPLE completed’

# Step 5: Diamond functional annotation

echo ‘Step 5: Diamond functional annotation...’

# Merge gene sequences across all samples

# Annotate against Nr database

echo ‘Annotating Nr database...’

# Annotate against KEGG database

echo ‘Annotating KEGG database...’

# Annotate against eggNOG database

echo ‘Annotating eggNOG database...’

# Annotate against CAZy database

echo ‘Annotating CAZy database...’

# Annotate against PHI database

echo ‘Annotating PHI database...’

# Annotate against VFDB database

echo ‘Annotating VFDB database...’

# Annotate against CARD database

echo ‘Annotating CARD database...’

Step 6: Generate summary report of results

echo ‘Step 6: Generating summary report of results...’

echo ‘Metagenomic analysis summary’ >> $SUMMARY_FILE

echo ‘Generated at: $(date)’ >> $SUMMARY_FILE

echo ‘Project name: $PROJECT_NAME’ >> $SUMMARY_FILE

echo ‘Number of samples: ${#UNIQUE_SAMPLES[@]}’ >> $SUMMARY_FILE

echo ‘Sample list: ${UNIQUE_SAMPLES[*]}’ >> $SUMMARY_FILE

echo ‘’ >> $SUMMARY_FILE

# Add statistics for each sample

echo ‘Statistics for each sample:’ >> $SUMMARY_FILE

# Obtain raw read count

# Obtain cleaned read count

# Obtain contig count and N50

# Obtain predicted gene count

# Obtain alignment rate

# Write to summary

echo ‘Sample: $SAMPLE’ >> $SUMMARY_FILE

echo ‘ Raw reads: $RAW_READS’ >> $SUMMARY_FILE

echo ‘ Cleaned reads: $CLEAN_READS’ >> $SUMMARY_FILE

echo ‘ Contig count: $CONTIG_COUNT’ >> $SUMMARY_FILE

echo ‘ N50: $N50’ >> $SUMMARY_FILE

echo ‘ Number of predicted genes: $GENE_COUNT’ >> $SUMMARY_FILE

echo ‘ Mapping rate: $MAPPING_RATE’ >> $SUMMARY_FILE

echo ‘’ >> $SUMMARY_FILE

# Add annotation statistics

echo ‘Functional annotation statistics:’ >> $SUMMARY_FILE

echo ‘==========================================’ >> $SUMMARY_FILE

echo ‘Nr annotated genes: $(wc -l < $ANNOTATION_DIR/nr/nr_annotations.tsv)’ >> $SUMMARY_FILE

echo ‘KEGG annotated genes: $(wc -l < $ANNOTATION_DIR/kegg/kegg_annotations.tsv)’ >> $SUMMARY_FILE

echo ‘Number of eggNOG annotated genes: $(wc -l < $ANNOTATION_DIR/eggnog/eggnog_annotations.tsv)’ >> $SUMMARY_FILE

echo ‘CAZy annotated genes: $(wc -l < $ANNOTATION_DIR/cazy/cazy_annotations.tsv)’ >> $SUMMARY_FILE

echo ‘PHI annotated genes: $(wc -l < $ANNOTATION_DIR/phi/phi_annotations.tsv)’ >> $SUMMARY_FILE

echo ‘VFDB annotated genes: $(wc -l < $ANNOTATION_DIR/vfdb/vfdb_annotations.tsv)’ >> $SUMMARY_FILE

echo ‘CARD annotated genes: $(wc -l < $ANNOTATION_DIR/card/card_annotations.tsv)’ >> $SUMMARY_FILE

# Calculate total runtime

echo ‘Total runtime: $(($ELAPSED_TIME / 3600)) hours $((($ELAPSED_TIME % 3600) / 60)) minutes $(($ELAPSED_TIME % 60)) seconds’ >> $SUMMARY_FILE

echo ‘Analysis complete! Summary saved to: $SUMMARY_FILE’

# Step 7: Generate visualisation report (optional)

echo ‘Step 7: Generating visualisation report...’

# Insert code here to generate charts and visualisation reports

# e.g. using R or Python to produce statistical graphs

echo ‘Metagenomic analysis workflow fully completed!’

**R package data visualization and statistical analysis code**

# Install necessary R packages

# Load packages

# Set theme and colours

# Set random seed to ensure reproducible results

Data loading and preprocessing

# Read data

# Species abundance matrix (rows are species, columns are samples)

# Functional abundance matrix (e.g. KEGG, COG)

# Sample metadata

# Ensure data consistency

# Data preprocessing: Filter low-abundance species/functions

# Calculate average relative abundance for each species/function

# Retain rows with average relative abundance above threshold

# Data standardisation (relative abundance)

Visualisation of species composition analysis

# 1. Species composition stacked bar chart

# Calculate average abundance for each species and select top_n

# Extract data for top_n species

# Add remaining species as ‘Others’

# Add grouping information

# Convert data to long format

# Plot stacked bar chart

# Plot species composition diagram

# 2. Grouped Species Composition Box Plot

# Extract data for specific species

# Add grouping information

# Plot box plot

# Plot box plot for specific species

# Add other species to merge into ‘Others’ # Add grouping information # Convert data to long format # Plot stacked bar chart

Functional Component Analysis Visualisation

# 1. Functional Pathway Abundance Heatmap

# Calculate the average abundance for each function and select the top_n

# Extract data for the top_n functions

# Add grouping information for annotation

# Plot heatmap

# Plot functional heatmap

# 2. Comparison of Specific Functional Pathways Across Groups

# Extract data for specific functions

# Add grouping information

# Plot box plots

# Plot comparison diagram for specific functional pathways

Alpha Diversity Analysis Visualisation

# Calculate alpha diversity

# Convert to matrix format

# Compute diversity indices

# Calculate alpha diversity

# Add grouping information

# Visualise alpha diversity

# Convert data to long format

# Plot box plots

# Plot alpha diversity diagram

Beta Diversity Analysis Visualisation

# Calculate Beta Diversity

# Calculate Distance Matrix

# Perform PCoA Analysis

# Calculate Bray-Curtis Distance

# Perform PCoA Analysis

# Add Group Information

# Visualise PCoA Results

# Plot PCoA Plot

# PERMANOVA Analysis (Intergroup Difference Test)

# Perform PERMANOVA

# Calculate Beta Diversity # Calculate Distance Matrix # Perform PCoA Analysis # Calculate Bray-Curtis Distance # Perform PCoA Analysis # Add Group Information # Visualise PCoA Results # Plot PCoA Plot # PERMANOVA Analysis (Intergroup Difference Test)

Visualisation of Differential Abundance Analysis

# Performing differential abundance analysis using DESeq2

# Creating a DESeq2 object

# Setting the reference level (if applicable)

# Running the DESeq2 analysis

# Extracting results

# Executing differential abundance analysis

# Visualising differential analysis results

# Preparing data

# Plotting a volcano plot

# Plotting a Volcano Plot

# Plotting a Heatmap of Differentially Abundant Species

# Filtering Significantly Differentially Abundant Species

# Extracting Data for Significantly Differentially Abundant Species

# Adding Group Information for Annotation

# Plotting the Heatmap

# Plotting a Heatmap of Differentially Abundant Species

Visualising Functional Enrichment Analysis

# Functional Enrichment Analysis (using KEGG pathways as an example)

# Screening for Significantly Differing Species

# Obtaining Functional Annotations for These Species

# Assuming we have a pathway_annotation data frame containing species-to-KEGG pathway mappings

# Performing Enrichment Analysis

# Visualising Enrichment Results

# Selecting the Top_n Enriched Pathways

# Plotting a Bar Chart

# Plot enrichment analysis diagram

Correlation Analysis Visualisation

# Calculate correlations between species and environmental factors

# Compute correlation matrix

# Assuming we have environmental factor data

# Calculate correlations

# Visualise correlation heatmap

# Create correlation heatmap

# Add significance markers (if p-value matrix available)

# Plot correlation heatmap

# Mantel test (correlation between species composition and environmental factors)

# Calculate species distance matrix

# Calculate environmental distance matrix

# Perform Mantel test

# Perform Mantel test

Save results and graphics

# Save graphics

# Save statistical results

# Save PERMANOVA results

# Save session information (for reproducibility)

# Save results and graphics # Save statistics # Save PERMANOVA results # Save session information (for reproducibility)

**Application of FDR correction in metagenomic analysis**

# Simulate metagenomic species abundance data

# Generate species abundance matrix (using negative binomial distribution to simulate count data)

# Create grouping information (e.g. disease vs healthy)

# Simulate some species with genuinely differentially abundant

# Perform Wilcoxon signed-rank test (suitable for microbiome data)

# Calculate median fold change

# Apply FDR correction

# Create results data frame

# View number of significantly differentially abundant species

cat(‘Number of significantly differentially abundant species (FDR < 0.05):’, sum(da_results$significant), ‘\n’)

# Plot Manhattan plot displaying FDR-corrected results

# Filter and save significant results

# Simulate functional enrichment analysis results (e.g. KEGG pathway enrichment analysis)

# Generate simulated pathway enrichment results

# Randomly select some pathways as genuinely enriched pathways

# Make the p-values for these pathways smaller

# Apply FDR correction

# View number of significantly enriched pathways

cat(‘Number of significantly enriched pathways (FDR < 0.05):’, sum(pathway_results$significant), ‘\n’)

# Plot enrichment analysis results

# Save enrichment analysis results

**In Chinese**

*# 宏基因组完整分析流程*

*# 包含*

*# 设置参数*

*内存使用限制*

*# 数据库路径*

*# 创建目录结构*

*# 记录开始时间*

开始宏基因组分析流程

*# 获取样本列表*

*# 去重样本列表*

找到

个样本

*# 步骤1: FASTP质量控制*

echo "步骤1: FASTP质量控制..."

echo "处理样本: $SAMPLE"

echo "错误: 找不到样本 $SAMPLE 的读段文件"

*# 运行FASTP*

echo "样本 $SAMPLE 质量控制完成"

*# 步骤2: MEGAHIT组装*

echo "步骤2: MEGAHIT组装..."

echo "组装样本: $SAMPLE"

*# 运行MEGAHIT*

*# 重命名contigs文件以包含样本名*

echo "样本 $SAMPLE 组装完成"

*# 步骤3: MetaGeneMark基因预测*

echo "步骤3: MetaGeneMark基因预测..."

echo "预测样本: $SAMPLE 的基因"

*# 运行MetaGeneMark*

echo "样本 $SAMPLE 基因预测完成"

*# 步骤4: Bowtie2读段比对*

echo "步骤4: Bowtie2读段比对..."

echo "比对样本: $SAMPLE"

*# 为每个样本的contigs构建Bowtie2索引*

*# 运行Bowtie2比对*

*# 转换SAM为BAM并排序*

*# 创建BAM索引*

*# 生成比对统计*

echo "样本 $SAMPLE 比对完成"

*# 步骤5: Diamond功能注释*

echo "步骤5: Diamond功能注释..."

*# 合并所有样本的基因序列*

*# 对Nr数据库注释*

echo "注释Nr数据库..."

*# 对KEGG数据库注释*

echo "注释KEGG数据库..."

*# 对eggNOG数据库注释*

echo "注释eggNOG数据库..."

*# 对CAZy数据库注释*

echo "注释CAZy数据库..."

*# 对PHI数据库注释*

echo "注释PHI数据库..."

*# 对VFDB数据库注释*

echo "注释VFDB数据库..."

*# 对CARD数据库注释*

echo "注释CARD数据库..."

*# 步骤6: 生成结果摘要报告*

echo "步骤6: 生成结果摘要报告..."

echo "宏基因组分析结果摘要" > $SUMMARY_FILE

echo "生成时间: $(date)" >> $SUMMARY_FILE

echo "项目名称: $PROJECT_NAME" >> $SUMMARY_FILE

echo "样本数量: ${#UNIQUE_SAMPLES[@]}" >> $SUMMARY_FILE

echo "样本列表: ${UNIQUE_SAMPLES[*]}" >> $SUMMARY_FILE

echo "" >> $SUMMARY_FILE

*# 添加每个样本的统计信息*

echo "各样本统计信息:" >> $SUMMARY_FILE

*# 获取原始读段数量*

*# 获取清洗后读段数量*

*# 获取contig数量和N50*

*# 获取预测基因数量*

*# 获取比对率*

*# 写入摘要*

echo "样本: $SAMPLE" >> $SUMMARY_FILE

echo " 原始读段数: $RAW_READS" >> $SUMMARY_FILE

echo " 清洗后读段数: $CLEAN_READS" >> $SUMMARY_FILE

echo " Contig数量: $CONTIG_COUNT" >> $SUMMARY_FILE

echo " N50: $N50" >> $SUMMARY_FILE

echo " 预测基因数: $GENE_COUNT" >> $SUMMARY_FILE

echo " 比对率: $MAPPING_RATE" >> $SUMMARY_FILE

echo "" >> $SUMMARY_FILE

*# 添加注释统计*

echo "功能注释统计:" >> $SUMMARY_FILE

echo "==========================================" >> $SUMMARY_FILE

echo "Nr注释基因数: $(wc -l < $ANNOTATION_DIR/nr/nr_annotations.tsv)" >> $SUMMARY_FILE

echo "KEGG注释基因数: $(wc -l < $ANNOTATION_DIR/kegg/kegg_annotations.tsv)" >> $SUMMARY_FILE

echo "eggNOG注释基因数: $(wc -l < $ANNOTATION_DIR/eggnog/eggnog_annotations.tsv)" >> $SUMMARY_FILE

echo "CAZy注释基因数: $(wc -l < $ANNOTATION_DIR/cazy/cazy_annotations.tsv)" >> $SUMMARY_FILE

echo "PHI注释基因数: $(wc -l < $ANNOTATION_DIR/phi/phi_annotations.tsv)" >> $SUMMARY_FILE

echo "VFDB注释基因数: $(wc -l < $ANNOTATION_DIR/vfdb/vfdb_annotations.tsv)" >> $SUMMARY_FILE

echo "CARD注释基因数: $(wc -l < $ANNOTATION_DIR/card/card_annotations.tsv)" >> $SUMMARY_FILE

*# 计算总运行时间*

echo "总运行时间: $(($ELAPSED_TIME / 3600))小时$((($ELAPSED_TIME % 3600) / 60))分钟$(($ELAPSED_TIME % 60))秒" >> $SUMMARY_FILE

echo "分析完成! 结果摘要保存在: $SUMMARY_FILE"

*# 步骤7: 生成可视化报告 (可选)*

echo "步骤7: 生成可视化报告..."

*# 这里可以添加生成图表和可视化报告的代码*

*# 例如使用R或Python生成统计图表*

echo "宏基因组分析流程全部完成!"

**R package data visualization and statistical analysis code**

*# 安装必要的R包*

*# 加载包*

*# 设置主题和颜色*

*# 设置随机种子以确保结果可重现*

数据加载与预处理

*# 读取数据*

*# 物种丰度表（行是物种，列是样本）*

*# 功能丰度表（如KEGG、COG等）*

*# 样本元数据*

*# 确保数据一致性*

*# 数据预处理：过滤低丰度物种/功能*

*# 计算每个物种/功能的平均相对丰度*

*# 保留平均相对丰度大于阈值的行*

*# 数据标准化（相对丰度）*

物种组成分析可视化

*# 1. 物种组成堆叠柱状图*

*# 计算每个物种的平均丰度并选择前top_n个*

*# 提取前top_n个物种的数据*

*# 添加其他物种合并为"Others"*

*# 添加分组信息*

*# 转换数据为长格式*

*# 绘制堆叠柱状图*

*# 绘制物种组成图*

*# 2. 分组物种组成箱线图*

*# 提取特定物种的数据*

*# 添加分组信息*

*# 绘制箱线图*

*# 绘制特定物种的箱线图*

功能组成分析可视化

*# 1. 功能通路丰度热图*

*# 计算每个功能的平均丰度并选择前top_n个*

*# 提取前top_n个功能的数据*

*# 添加分组信息用于注释*

*# 绘制热图*

*# 绘制功能热图*

*# 2. 特定功能通路在不同分组中的比较*

*# 提取特定功能的数据*

*# 添加分组信息*

*# 绘制箱线图*

*# 绘制特定功能通路的比较图*

Alpha多样性分析可视化

*# 计算Alpha多样性*

*# 转换为矩阵*

*# 计算多样性指数*

*# 计算Alpha多样性*

*# 添加分组信息*

*# 可视化Alpha多样性*

*# 转换数据为长格式*

*# 绘制箱线图*

*# 绘制Alpha多样性图*

Beta多样性分析可视化

*# 计算Beta多样性*

*# 计算距离矩阵*

*# 执行PCoA分析*

*# 计算Bray-Curtis距离*

*# 执行PCoA分析*

*# 添加分组信息*

*# 可视化PCoA结果*

*# 绘制PCoA图*

*# PERMANOVA分析（组间差异检验）*

*# 执行PERMANOVA*

差异丰度分析可视化

*# 使用DESeq2进行差异丰度分析*

*# 创建DESeq2对象*

*# 设置参考水平（如果有的话）*

*# 运行DESeq2分析*

*# 提取结果*

*# 执行差异丰度分析*

*# 可视化差异分析结果*

*# 准备数据*

*# 绘制火山图*

*# 绘制火山图*

*# 绘制差异物种热图*

*# 筛选显著差异的物种*

*# 提取显著差异物种的数据*

*# 添加分组信息用于注释*

*# 绘制热图*

*# 绘制差异物种热图*

功能富集分析可视化

*# 功能富集分析（以KEGG通路为例）*

*# 筛选显著差异的物种*

*# 获取这些物种的功能注释*

*# 假设我们有 pathway_annotation 数据框，包含物种到KEGG通路的映射*

*# 执行富集分析*

*# 可视化富集结果*

*# 选择前top_n个富集通路*

*# 绘制条形图*

*# 绘制富集分析图*

相关性分析可视化

*# 计算物种与环境因子的相关性*

*# 计算相关性矩阵*

*# 假设我们有环境因子数据*

*# 计算相关性*

*# 可视化相关性热图*

*# 创建相关性热图*

*# 添加显著性标记（如果有p值矩阵）*

*# 绘制相关性热图*

*# Mantel检验（物种组成与环境因子的相关性）*

*# 计算物种距离矩阵*

*# 计算环境距离矩阵*

*# 执行Mantel检验*

*# 执行Mantel检验*

保存结果和图形

*# 保存图形*

*# 保存统计结果*

*# 保存PERMANOVA结果*

*# 保存会话信息（便于复现）*

**Application of FDR correction in metagenomic analysis**

*# 模拟宏基因组物种丰度数据*

*# 生成物种丰度矩阵（使用负二项分布模拟计数数据）*

*# 创建分组信息（例如疾病vs健康）*

*# 模拟一些真正差异丰度的物种*

*# 执行Wilcoxon秩和检验（适用于微生物组数据）*

*# 计算中位数折叠变化*

*# 应用FDR校正*

*# 创建结果数据框*

*# 查看显著差异丰度物种数量*

cat("显著差异丰度物种数量 (FDR < 0.05):", sum(da_results$significant), "\n")

*# 绘制曼哈顿图展示FDR校正结果*

*# 筛选并保存显著结果*

*# 模拟功能富集分析结果（如KEGG通路富集分析）*

*# 生成模拟通路富集结果*

*# 随机选择一些通路作为真正富集的通路*

*# 使这些通路的p值更小*

*# 应用FDR校正*

*# 查看显著富集通路数量*

cat("显著富集通路数量 (FDR < 0.05):", sum(pathway_results$significant), "\n")

*# 绘制富集分析结果图*

*# 保存富集分析结果*
